# Novel Transcriptomic Interactomes of Noncoding RNAs in the Heart under Altered Thyroid Hormonal States

**DOI:** 10.3390/ijms24076560

**Published:** 2023-03-31

**Authors:** Viswanathan Rajagopalan, Sankalpa Chakraborty, Richard Lin

**Affiliations:** 1 New York Institute of Technology College of Osteopathic Medicine, Arkansas State University, Jonesboro, AR 72401, USA; 2 Molecular Biosciences Graduate Program, College of Sciences and Mathematics, Arkansas State University, Jonesboro, AR 72401, USA; sankalpa.chakrabo@smail.astate.edu; 3 Arkansas Biosciences Institute, Jonesboro, AR 72401, USA

**Keywords:** heart, noncoding RNAs, transcriptome, interactome, thyroid hormones, cancer

## Abstract

Noncoding RNAs are emerging as vital players in cardiovascular diseases. Thyroid hormones (THs) are crucial for cardiovascular survival; however, correction of systemic hypothyroidism (low serum THs) may not improve cardiac tissue-level hypothyroidism or cardiac function. Mechanistically, the understanding of noncoding transcriptomic interactions influencing TH-mediated cardiac effects is unclear. Adult C57BL/6J mixed-sex mice were randomized into Control, Hypothyroid (HypoTH), Hyperthyroid (HyperTH), and HypoTH-Triiodothyronine restoration groups. Physiological, morphological, biochemical, molecular, and whole transcriptomic studies and appropriate statistical analyses were performed. HypoTH showed significant atrophy, depressed cardiac function, and decreased serum THs versus controls, and Triiodothyronine supplementation restored them. HyperTH significantly increased serum THs with hypertrophy. Real-time PCR showed significantly altered inflammatory and immune lncRNAs. The transcriptomic sequencing revealed significant differential expressions of lncRNAs, miRNAs, and mRNAs. Eleven novel circRNAs significantly decreased with increased THs. Multiple pathways were GO-/KEGG-enriched, including cardiac, thyroid, cancer, mitochondrial, inflammatory, adrenergic, metabolic, immune-mediated, vesicular, etc. We also uncovered significant novel co-expression and interactions of lncRNA-miRNA, lncRNA-miRNA-mRNA, lncRNA-mRNA, circRNA-miRNA, and miRNA-mRNA, and splicing events. This includes a novel pathway by which the predominant cardiac TH receptor alpha may interact with specific lncRNAs and miRNAs. This is the first study reporting a comprehensive transcriptome-wide interactome in the cardiac–thyroid axis.

## 1. Introduction

The protein-coding genes represent only about 2–5% of the entire mammalian genome. The majority of the remainder of the genome (noncoding) are actively transcribed [1,2]. Noncoding RNAs are emerging as critical regulators in the development and progression of cardiovascular disorders [3,4,5]. Understanding their roles could lead to better therapeutic and diagnostic opportunities. Multiple nucleic acid-based therapies have been approved by the Food and Drug Administration [6]. More clinical trials are ongoing, especially focusing on noncoding RNAs. Noncoding RNAs can be broadly classified into long and short based on their lengths.

Human long noncoding RNA (lncRNA; >200 nucleotides) genes outnumber human protein-coding genes by about 4.8-fold. Mouse lncRNA genes outnumber their respective protein-coding genes by about 4-fold (NONCODE [7]). LncRNAs play key roles in transcriptional and post-transcriptional gene regulation, modulation of alternative splicing and transcription, epigenetic modifications, interactions with messenger RNAs (mRNAs) and proteins in the cytoplasm to regulate gene expression, serving as biomarkers, etc. [8]. It has also been postulated that the majority of the lncRNAs might be functionally relevant, although heterogeneous in their mode of operation [9]. They undergo splicing of multi-exonic transcripts and may be found anywhere in the cell (with many of them in the nucleus) [2]. LncRNAs can functionally operate even at lower copy numbers [10].

While lncRNAs are linear, circular RNAs (CircRNAs) are covalently closed loop RNA molecules and are generated by back-splicing of exons and/or intron(s) [11,12]. Given the circularized structures, these molecules have greater stability than linear RNA and are protected from degradation by exonucleases. Their sizes can range from <100 nucleotides to multiple kilobases, with a median length of ~500–700 nucleotides. CircRNAs can also serve as microRNA sponges and biomarkers, regulate transcription, interact with RNA-binding proteins, etc. In the heart, ~9% of the expressed genes produce circRNAs [13], and they can be derived from protein-coding transcripts as well as non-coding transcripts, such as lncRNAs. CircRNAs and epigenomic factors can be conserved and expressed in human, mouse, and pig hearts [14,15].

Among the short noncoding RNAs, microRNAs (miRNAs/miRs) have been explored since the turn of the century for their numerous pathophysiological roles [16,17,18]. They are single-stranded and 21–23 base pairs long. Through a Drosha- and DICER-dependent mechanism in the cytosol, mature miRNAs are processed from pre-miRNAs and pri-miRNAs [19]. By complementary base-pairing to 5′ or 3′ untranslated regions of mRNAs, or sometimes within the gene body, miRNAs suppress gene expression [20]. However, miRNAs can also activate gene expression [21,22]. MiRNAs are well-studied regulators of cardiovascular structure, function, development, and disease [23,24].

Numerous clinical studies have shown that abnormalities associated with thyroid hormones (THs) can lead to detrimental effects on the heart [25,26,27,28,29]. Both hypothyroidism (insufficient/low TH levels; HypoTH) and hyperthyroidism (excessive/high TH levels; HyperTH) can affect the cardiovascular system in a deleterious manner [30]. The common complications include arrhythmias, cardiac dilatation, heart failure, etc. At the fundamental level, the THs (3,5,3′,5′-tetraiodothyronine, T4 [thyroxine], and 3,4,3′-triiodothyronine, T3) are involved in the regulation of cardiomyocyte growth, heart rate, contractile and relaxational properties, hypertrophy, fibrosis, stroke volume, pulse pressure, vascular resistance, etc. [31,32,33,34,35,36,37]. Our past preclinical studies have shown that optimal treatment with T3, the active form of TH, can offer safe and beneficial cardioprotective effects in myocardial infarction, ischemia-reperfusion injury, diabetes mellitus, etc. [38,39,40,41]. Some clinical studies also support the beneficial effects of TH-based therapy [42,43]. However, serum T3 levels may not accurately reflect cardiac tissue T3 levels. Correction/reversibility of systemic HypoTH does not necessarily improve cardiac tissue HypoTH [44,45]. Thus, a better understanding of the molecular mechanisms and heart-specific targets in the cardiac-TH signaling pathway could pave way for better diagnostics and therapeutics. Among cancer survivors, cancer treatment-associated cardiotoxicity is a leading cause of treatment-related morbidity and mortality. In addition, studies have reported alteration in TH levels associated with cancer chemotherapy [46,47].

Studies have also shown functional involvement of miRNAs in the heart associated with thyroid dysfunction [48,49,50,51]. A recent study from our group highlighted that the T3-induced protection from cardiac decompensation secondary to acute severe caloric restriction is partly mediated by lncRNAs [52]. Furthermore, studies have also revealed interactions among different types of noncoding RNAs and with proteins. Such interactions have been identified in both cardiovascular disorders and cancer [53]. Functional interactions could evoke responses of pathophysiological significance [4,8,54,55,56,57]. However, the role of circRNAs and a comprehensive role of lncRNAs in thyroid alterations-induced cardiac dysfunction is unknown. In addition, a detailed analysis of interactions involved in the cardiac transcriptome of major noncoding RNAs following alterations in TH levels is lacking and this study aims to address the gap.

We hypothesized a complex network of noncoding and coding RNA interactions in the cardiac TH pathway. We studied the transcriptome and the networks using established models of HypoTH and HyperTH. The findings revealed several novel targets and a comprehensive network of interactions among the lncRNAs, circRNAs and miRNAs with the mRNAs in the heart facing deficient, excessive, or restored THs.

## 2. Results

### 2.1. Oral T3 Supplementation Significantly Restores Serum TH Levels in HypoTH

**Figure 1:**
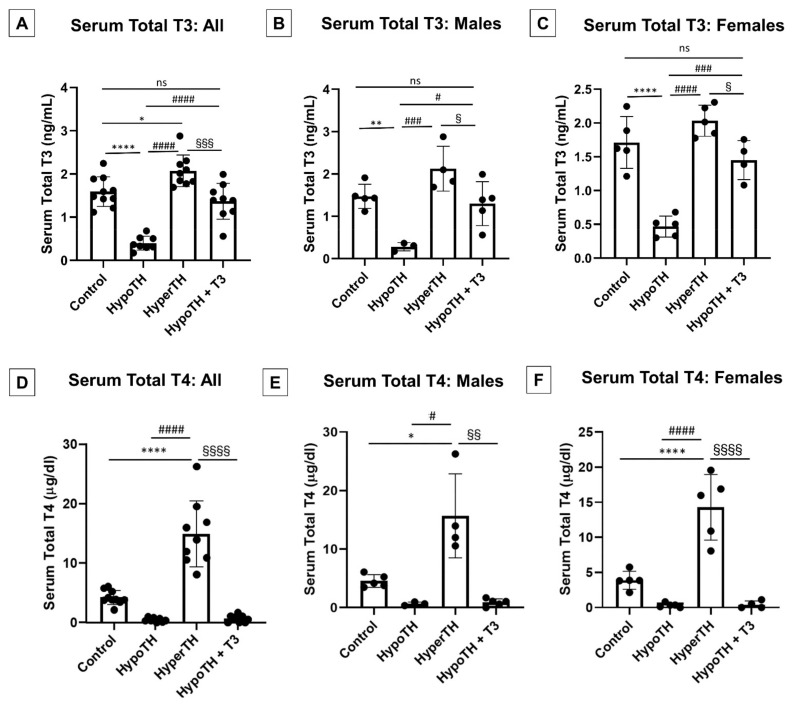
**Serum levels under altered thyroid hormonal states: Serum levels of** (**A**–**C**) **total T3** and (**D**–**F**) **total T4** showed reductions in HypoTH, increases in HyperTH, restored T3 levels, T4 feedback inhibition and similar trends in both the sexes. (**A**,**D**) All mice; (**B**,**E**) Male mice; (**C**,**F**) Female mice; All values are means ± standard deviation; *n* = 8–10 per group. TH: thyroid hormone; HyperTH: Hyperthyroidism; HypoTH: Hypothyroidism; T4: Thyroxine; T3: Triiodothyronine; * *p* < 0.05, ** *p* < 0.01, **** *p* < 0.0001 vs. control group; # *p*< 0.05, ### *p* < 0.001, #### *p* < 0.0001 vs. HypoTH group; § *p* < 0.05, §§ *p* < 0.01, §§§ *p* < 0.001, §§§§ *p* < 0.0001 vs. HyperTH group; ns = not significant.

The TH status was confirmed by assessing the serum T3 and T4 levels in all the mice (Figure 1). Compared to the control mixed-sex mice, the results showed a significant decrease in the total T3 and total T4 levels in the HypoTH mice. In contrast, the total T3 and T4 levels were significantly increased in the HyperTH group. Importantly, oral T3 treatment restored the total T3 levels in HypoTH mice to a systemic euthyroid state. In addition, the total T4 levels remained low in HypoTH mice following oral T3 treatment, thus demonstrating the anticipated feedback inhibition mechanism [38,39]. These also validate successful and optimal T3 supplementation therapy. Furthermore, the directions of changes were similar and predominantly overall significant when we separated the males and females for both the TH levels (Figure 1B,C,E,F).

### 2.2. Morphometrics

**Figure 2:**
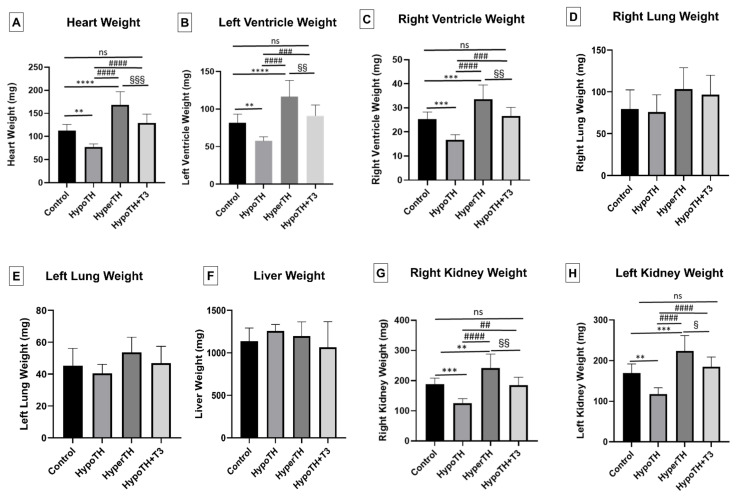
**Morphometrics under altered TH states (all mice):** Alterations in (**A**) Heart, (**B**) Left Ventricle, (**C**) Right Ventricle, (**D**) Right Lung, (**E**) Left Lung, (**F**) Liver, (**G**) Right Kidney, (**H**) Left Kidney weights of mixed sex mice when subjected to HypoTH, HyperTH, and restoration in the HypoTH group following oral T3. All values are means ± standard deviation; *n* = 8–10 per group. TH–thyroid hormone; HyperTH: Hyperthyroidism; HypoTH: Hypothyroidism; T3: Triiodothyronine; ** *p* < 0.01, *** *p* < 0.001, **** *p* < 0.0001 vs. controls; ## *p* < 0.01, ### *p* < 0.001, #### *p* < 0.0001 vs. HypoTH; § *p* < 0.05, §§ *p* < 0.01, §§§ *p* < 0.001 vs. HyperTH; ns: not significant.

In age-matched mixed-sex mice, results showed that the HypoTH significantly (Figure 2; *p* < 0.05) decreased the heart, LV, and right and left kidney weights compared to the controls. HyperTH induction resulted in a significant increase in heart, LV, RV, and kidney weights (both left and right) compared to both the control and HypoTH mice. Oral T3 treatment administered to HypoTH mice significantly restored the heart, LV, RV, and kidney weights (both left and right) comparable to the controls (ns). They were also significantly higher than HypoTH and lower than the HyperTH groups. Analysis of these gravimetric data by separating male (Supplementary Figure S1) and female mice (Supplementary Figure S2) showed predominantly similar and significant changes.

### 2.3. Echocardiogram

Cardiac ultrasound showed that interventricular septal thickness, and to a partial extent, LV (left ventricular/ventricle) posterior wall thickness, were diminished in HypoTH and both were significantly increased in HyperTH and HypoTH + T3 groups, more clearly in systole. These support the morphometric changes presented above. In addition, heart rate and fractional shortening were also significantly diminished in HypoTH and were significantly improved in the HyperTH and HypoTH + T3 groups. LV internal diameter significantly improved in systole only with the T3 treatment group. The results are presented in Table 1.

### 2.4. Significant Impairment in Cardiac Inflammatory lncRNA Expression under Altered TH Conditions

As a starting point, we began investigating the involvement of lncRNAs in inflammatory responses and immune mechanisms, which are associated with both TH dysfunction and HF. Among the 86 lncRNAs studied, a scatter plot of quantitative real-time PCR (qPCR) showed several lncRNAs significantly upregulated (red) and downregulated (green) under hyperthyroid and hypothyroid conditions (*p* < 0.05; Supplementary Figure S3). Dotted lines indicate fold regulation threshold (+/−1.5). To evaluate the transcriptome in further detail, we pursued a comprehensive transcriptomic sequencing approach.

### 2.5. Whole Transcriptome Analysis

Global transcriptome analysis was performed to identify profiles of major non-coding transcripts (lncRNAs, circRNAs, miRNAs) and coding transcripts (mRNAs) that are altered in response to distinct altered TH states.

#### 2.5.1. LncRNA Analyses

**Figure 3:**
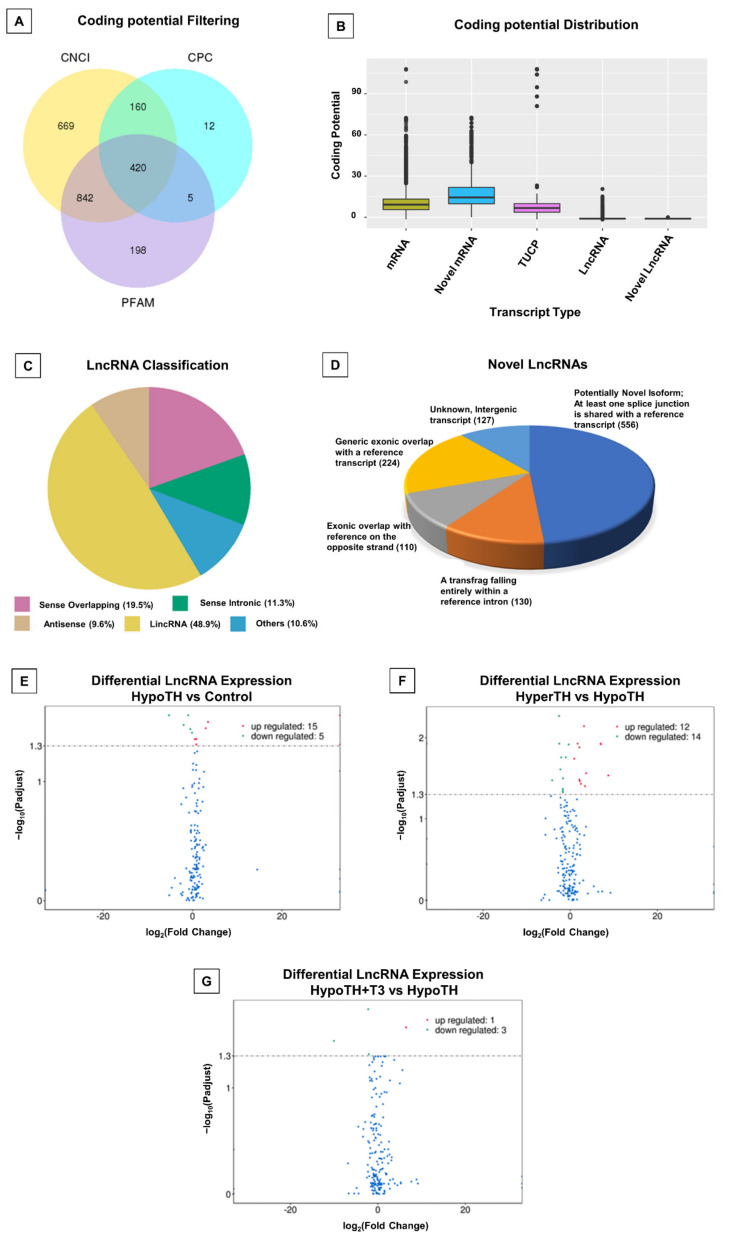
Novel and significant lncRNA alterations in TH dysfunction and restoration: (**A**) Coding potential Filtering: Numbers in each circle and overlap represent the respective total and shared number of predicted noncoding transcripts; (**B**) Coding potential distribution; (**C**) LncRNA classification; (**D**) Novel lncRNAs and different subtypes; (**E**–**G**) Differential LncRNA Expression Analysis of (**E**) HypoTH vs. Control (**F**) HyperTH vs. HypoTH and (**G**) HypoTH + T3 vs. HypoTH groups. Targets with adjusted/corrected *p* < 0.05 were considered statistically significant; LncRNA-Long noncoding RNA; CNCI: Coding-Non-Coding Index; CPC: Coding potential Calculator; TUCP: Transcripts of uncertain coding potential; HyperTH: Hyperthyroidism; HypoTH: Hypothyroidism; T3: Triiodothyronine.

The lncRNA quality control data for all the samples are shown in Supplementary Table S1. We first studied the lack of coding potential of lncRNAs and their sequence conservation with known proteins using (i) Coding-Non-Coding Index (CNCI; vertebrate), (ii) Coding Potential Calculator (CPC), and (iii) Pfam Analysis. The Venn diagram in Figure 3A shows lncRNAs detected by the individual tools and overlapping in more than one tool. Transcripts predicted with coding potential by either/all of the three tools being filtered out, and those without coding potential were used as the candidate set of lncRNAs (Figure 3B). The lncRNAs were classified as long intergenic noncoding RNAs (48.9%), sense-overlapping (19.5%), antisense (9.6%), sense_intronic (11.3%), etc. (Figure 3C). We have also identified a total of 1147 novel lncRNA transcripts (Figure 3D). The list with details is provided in Supplementary File S1.

**Significant changes in cardiac lncRNAs differentially expressed under altered TH states:** We found 12 upregulated and 2 downregulated lncRNAs (at least ±1.5-fold; adjusted *p* < 0.05) between HypoTH and controls (Figure 3E). Following the restoration of T3 levels in HypoTH mice, three lncRNAs were downregulated (*Fam120aos*, *9630028I04Rik* and *Gm31678*) and one (*Gm52092*) was upregulated (Figure 3G). Between the HyperTH and HypoTH groups, 12 were downregulated and 11 were upregulated (Figure 3F; at least ±1.5-fold; adjusted *p* < 0.05). Furthermore, compared to controls, the lncRNA, *Gm42067* decreased in HypoTH and increased in HyperTH. On the other hand, *Synpo2* and *mt-Rnr2* were high in HypoTH and low in control and HyperTH. While *Fam120aos* increased in HypoTH and decreased in both HyperTH and HypoTH + T3, *Gm52092* decreased in HypoTH and increased in HyperTH and HypoTH + T3. Importantly, the statistically significant increase in the level of *Gm52092* in HypoTH + T3 was 1.5-fold lower than that of HyperTH. The complete list of these targets and details are presented in Supplementary Tables S2–S4. The DE (differentially expressed or differential expression) genes heatmap is provided in Supplementary Figure S4A.

**Figure 4:**
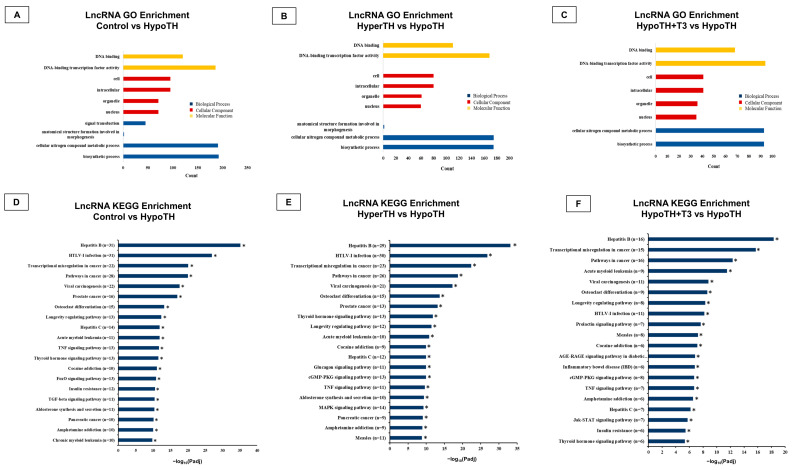
**GO and KEGG analyses of lncRNAs revealed significant enrichment:** Significantly enriched (**A**–**C**) GO terms (*p* < 0.05) in (**A**) Control vs. HypoTH, (**B**) HyperTH vs. HypoTH and (**C**) HypoTH + T3 vs. HypoTH groups and (**D**–**F**) Top 20 KEGG pathways associated with differentially expressed genes compared with background in (**D**) Control vs. HypoTH, (**E**) HyperTH vs. HypoTH and (**F**) HypoTH + T3 vs. HypoTH groups. Statistically significant KEGG enrichments are marked with asterisks; GO: Gene Ontology; KEGG: Kyoto Encyclopedia of Genes and Genomes; HyperTH: Hyperthyroidism; HypoTH: Hypothyroidism; T3: Triiodothyronine; *p*-adj—adjusted/corrected; * *p* < 0.05.

LncRNA Enrichment analyses: The Gene Ontology (GO) analysis showed significantly enriched GO terms in source genes compared to the reference genes’ background (Figure 4A–C; *p* < 0.05). The three ontologies, molecular function (MF), cellular component (CC), and biological process (BP), were studied. In all group comparisons, two common pathways showed significant enrichment in the MFs, DNA binding and DNA binding transcription factor activity. Regarding BP enrichment, the common significant processes identified in all the groups were the biosynthetic process and cellular nitrogen compound metabolic process. Additionally, both Control vs. HypoTH and HyperTH vs. HypoTH + T3 comparisons showed significant BP enrichment in anatomical structure formation involved in morphogenesis. The control vs. HypoTH group also showed significant BP enrichment in signal transduction. Among all the groups, four CC GO enriched hits were nucleus, organelle, cell, and intracellular. Complete results are provided in Supplementary File S2.

The Kyoto Encyclopedia of Genes and Genomes (KEGG) Pathway enrichment analysis for lncRNA identified significantly enriched pathways associated with DE lncRNA host genes compared with the whole genome background. Based on the alterations in DE analysis (Figure 3E–G), the three comparisons (Control vs. HypoTH, HyperTH vs. HypoTH and HypoTH + T3 vs. HypoTH) showed significant hits in KEGG enrichment (Figure 4B). Among the numerous pathways significantly altered in the heart, TH signaling pathway, TH synthesis pathway and thyroid cancer were notable. Six common TH signaling pathway genes were identified as potential lncRNA targets: *Foxo1*, *GATA4*, *Rxrb*, TH receptor alpha (*Thra*), TH receptor beta (*Thrb*), and *STAT1*. Moreover, in all three group comparisons, many of the top, statistically significant hits were cancer-associated pathways. Among others, *E2f1*, *E2f3*, *Kif13a*, *Rxra*, *Rxrb*, and *Rxrg* were the most common and prominent genes that emerged in our analysis associated with multiple cancer-related pathways. The complete KEGG enrichment analysis is included in Supplementary File S3.

**LncRNA Alternative Splicing:** We also investigated alternative splicing (AS) with both reads on target and reads span splicing junctions using rMATS (replicate multivariate analysis of transcript splicing). The five AS types studied were Skipped exon (SE), Alternative 5′ splice site (A5SS), Alternative 3′ splice site (A3SS), Mutually exclusive exon (MXE), and Retained intron (RI). We found numerous AS events in the group comparisons and the threshold of differential AS analysis was set to FDR < 0.05. All relevant results are listed in Supplementary File S4.

#### 2.5.2. CircRNA Analyses

The circRNA quality control summary for all the groups is provided in Supplementary Table S1.

**Figure 5:**
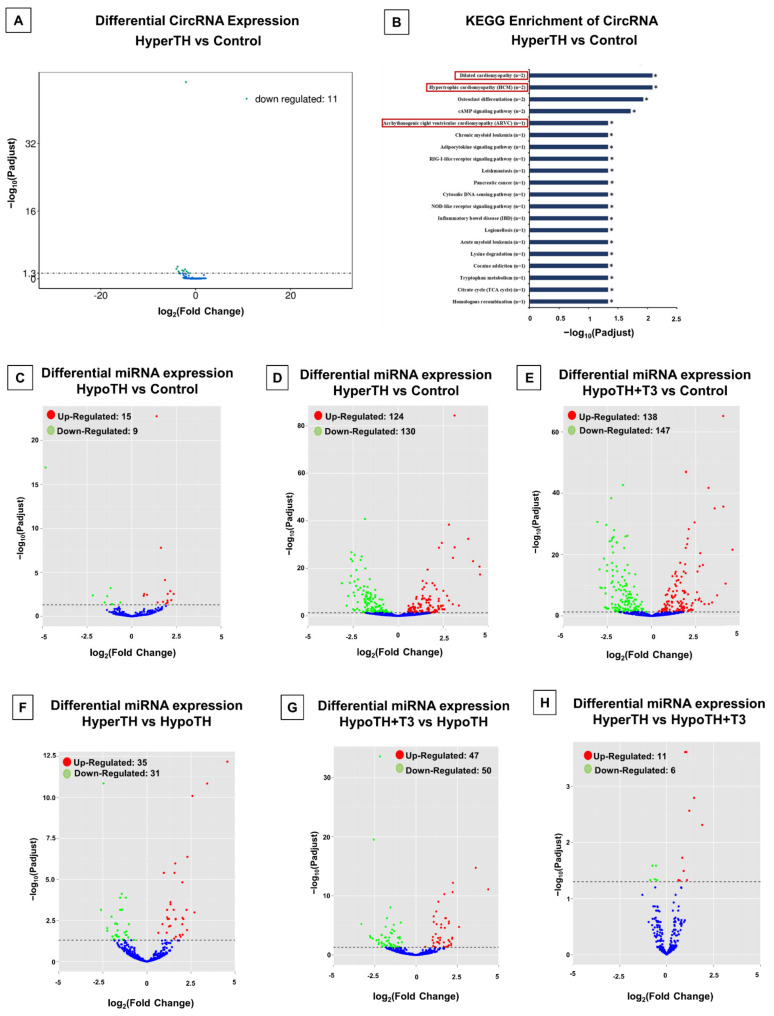
Differential Expression Analyses of CircRNAs and miRNAs. (**A**) Comparison of HyperTH (Hyperthyroid) and Control groups revealed 11 CircRNAs, which were significantly differentially expressed. (**B**) KEGG enrichment analysis of top 20 pathways with significantly altered circRNA targets between HyperTH and Controls. Statistically significant KEGG enrichments are marked with asterisks. Cardiac disease-related pathways are highlighted in red boxes. (**C**–**H**) Differential expression analysis of miRNAs in (**C**) HypoTH vs. Control (**D**) HyperTH vs. Control (**E**) HypoTH + T3 vs. Control (**F**) HyperTH vs. HypoTH (**G**) HypoTH + T3 vs. HypoTH and (**H**) HyperTH vs. HypoTH + T3 groups. For all analyses, the targets were considered statistically significant with *p*-adj(corr) < 0.05.

**CircRNA Differential Expression:** Among all the comparisons to identify DE circRNAs, only the HyperTH vs. Control comparison yielded statistically significant results (Figure 5A). We found that 11 circRNAs (*mmu_circ_4456*, *mmu_circ_5616*, *mmu_circ_2471*, *mmu_circ_3252*, *mmu_circ_1539*, *mmu_circ_799*, *mmu_circ_2045*, *mmu_circ_1548*, *mmu_circ_2258*, *mmu_circ_960*, *mmu_circ_384*) were downregulated. The complete DE analysis result is provided in Supplementary Table S5 (with at least +/−1.5-fold change), and the DE genes heatmap is provided in Supplementary Figure S4B.

**Alternative backsplicing of circRNA:** Circular RNAs (circRNAs) are synthesized from pre-mRNAs through backsplicing. We employed CIRCexplorer2 to detect alternative back-spliced circRNAs. Besides circRNAs with annotated exons, it could identify novel circRNA-specific exons, which are not expressed in linear RNAs. We explored the two types of alternative back-splicing—5′ and 3′. Complete results for alternative backsplicing of the DE circRNAs (including chromosomal location, strand, isoform type, etc.) are provided in Supplementary File S5.

**CircRNA Enrichment analysis:** GO and KEGG enrichment analyses were performed with the host genes of DE circRNAs. While GO analysis did not show significant enrichment, KEGG analysis had significantly enriched pathways for the HyperTH vs. control group comparison (Figure 5B). Forty-four enriched pathways include four cardiac-associated pathways: dilated cardiomyopathy (DCM), hypertrophic cardiomyopathy (HCM), arrhythmogenic right ventricular cardiomyopathy (ARVC), and cardiac muscle contraction. One common host gene association emerged from all the cardiac disease and cardiac muscle contraction pathways: Ryanodine receptor 2 (*Ryr2*). Significant cancer-associated pathways include acute myeloid leukemia, chronic myeloid leukemia, pancreatic cancer, prostate cancer, and small cell lung cancer. Among these cancer-associated pathways, interestingly, only one gene, Nuclear factor of kappa light polypeptide gene enhancer in B cells 1, p105 (*Nfkb1*), was found to be involved in all aforementioned types of cancer and cancer-associated pathways. In addition, this gene was associated with the most number of pathways enriched in this analysis. The complete result is presented in Supplementary File S6.

#### 2.5.3. miRNA Analyses

A summary of various types of small RNAs is listed in Supplementary Table S6. The small RNA quality control summary for all the groups is provided in Supplementary Table S7. The major small RNA, miRNA, is mainly studied here.

**miRNA Differential expression:** DE analysis of miRNAs revealed statistically significant alterations in all the group comparisons (Figure 5C–H). The greatest number of alterations were seen when controls were compared with HyperTH or HypoTH + T3. miRNAs that were increased in HypoTH compared to controls and were decreased in HyperTH and HypoTH + T3 include *mmu-miR-466i-5p*, *mmu-miR-3470b*, and *mmu-miR-6538*. We have also identified miRNAs that decreased in HypoTH compared to control and were increased in HyperTH and HypoTH + T3, including *mmu-miR-208a-3p* and *mmu-miR-208a-5p*. Moreover, there were DE miRNAs that were increased in HypoTH compared to the control group and were restored in HypoTH + T3 that include *mmu-miR-1195*, *mmu-miR-5126*, *mmu-miR-200a-3P*, and *mmu-miR-200b-3p*. A complete list of differentially regulated miRNAs is listed in Supplementary File S7 (with at least +/−1.5-fold change), and the DE genes heatmap is provided in Supplementary Figure S4C.

**miRNA Enrichment analyses:** All six group comparisons showed statistically significant GO enrichment. Notably, the HypoTH vs. Control group comparison showed significant enrichment in 145 BP, 16 CC, and 137 MF ontologies. Following T3 treatment in HypoTH, compared to the untreated HypoTH group, 323 GO enrichment items (143 BP, 28 CC, 152 MF) were identified. Furthermore, the HyperTH vs. HypoTH comparison yielded 306 GO enrichment hits (137 BP, 22 CC, 147 MF). The top 20 hits for all GO comparisons for all the groupwise comparisons are provided in Supplementary Figure S5. In addition, the complete list of statistically significant GO enrichments in all groupwise comparisons is provided in Supplementary File S8.

We found statistically significant KEGG enrichment pathways for all the six group comparisons. Notably, there were 233 KEGG enrichment pathways for HypoTH vs. Control, 263 for HypoTH + T3 vs. HypoTH, and 260 for HyperTH vs. HypoTH. Many of the significant KEGG-enriched pathways were similar among different group comparisons and there were multiple common TH-associated and cardiac pathways, as seen in lncRNA KEGG enrichment (Supplementary File S3). These include the TH signaling pathway, TH synthesis, Adrenergic signaling in cardiomyocytes, Vascular smooth muscle contraction, Cardiac muscle contraction, DCM, ARVC, and HCM. In addition, multiple common TH signaling and cardiac disease or functional pathway genes were identified as miRNA host genes within all groupwise comparisons with the HypoTH group. This includes *Thra*, *Thrb*, Deiodinase 3 (*Dio3*), *Dio2*, *Dio1*, solute carrier family 16 member 10 (*Slc16a10*; *MCT10*), Sarco(endo)plasmic reticulum calcium-ATPase 2 (*SERCA2*; *Atp2a2*), Phospholamban (*Pln*), sodium/potassium-ATPase subunits alpha-1 and beta-2 (*Atp1a1* and *Atp1b2*), Sodium/Calcium Exchanger 1 (*NCX1*; *Slc8a1*), and Ryanodine receptor 2 (*Ryr2*). Furthermore, many cancer-associated pathways were identified, including, thyroid cancer, endometrial cancer, prostate cancer, pathways involved in transcriptional misregulation in cancer, viral carcinogenesis, acute myeloid leukemia, pancreatic cancer, and small cell and non-small cell lung cancer. The complete KEGG enrichment analysis is provided in Supplementary File S9.

**miRNA base editing:** We also studied base editing to investigate possible editing in miRNA nucleotides at 100% base edit read percentage [58]. We found a trend in the increase in the number of variants from U to G in the HypoTH group compared to the controls. In addition, the HyperTH and HypoTH + T3 groups tended to decrease in the number of variants from A to G compared to the controls.

#### 2.5.4. mRNA Analyses

**Figure 6:**
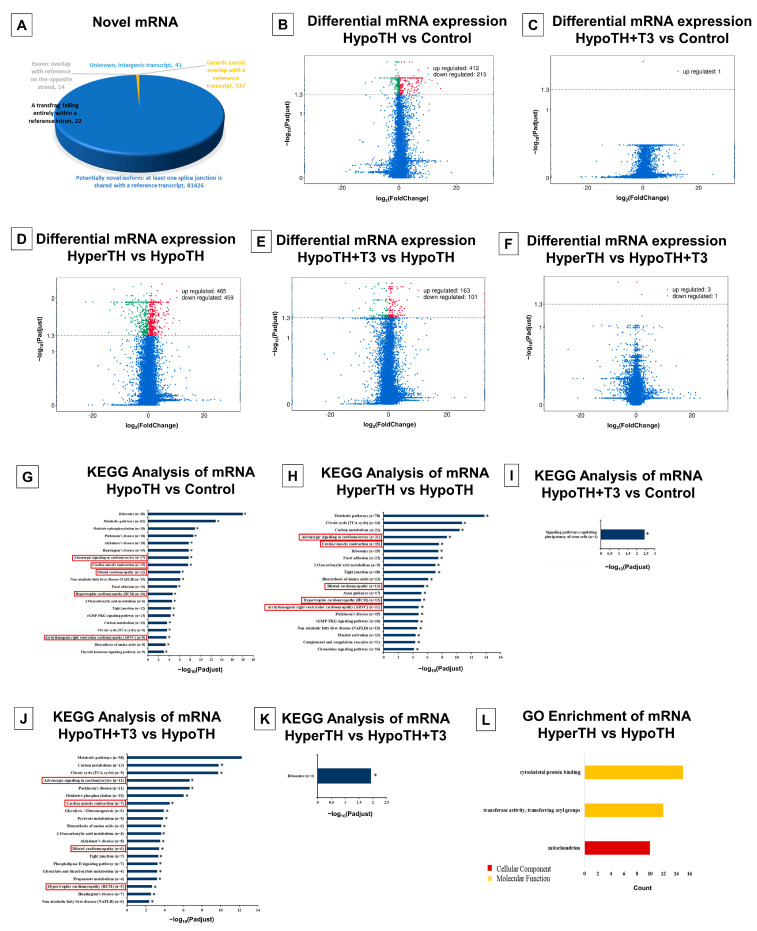
Novel and significant mRNA alterations and enrichments in TH dysfunction and restoration: (**A**) Novel mRNAs and different subtypes; (**B**–**F**) Significant differentially expressed mRNAs in (**B**) HypoTH vs. Control (**C**) HypoTH + T3 vs. Control (**D**) HyperTH vs. HypoTH (**E**) HypoTH + T3 vs. HypoTH (**F**) HyperTH vs. HypoTH + T3 groups; (**G**–**K**) KEGG enrichment analysis of pathways significantly altered mRNA targets in (**G**) HypoTH vs. Control (**H**) HyperTH vs. HypoTH (**I**) HypoTH + T3 vs. Control (**J**) HypoTH + T3 vs. HypoTH (**K**) HyperTH vs. HypoTH + T3 groups; (**L**) GO enrichment analysis of significantly altered mRNAs. Cardiac disease-associated pathways are highlighted in red boxes. Statistically significant KEGG hits (top 20 for (**G**,**H**,**J**) are also marked with asterisks. Only GO hits with *p*-adj(corr) < 0.05 were included in the analysis.

Our mRNA sequencing analyses identified 82,040 novel mRNA transcripts in the mouse LV (Figure 6A). The complete novel mRNA list is provided in Supplementary File S10.

**Differential expression of mRNAs:** DE analysis of coding mRNA transcripts identified significantly altered mRNAs in all the group comparisons except HyperTH vs. Control group (Figure 6B–F and Supplementary File S11). The DE genes heatmap for mRNA is provided in Supplementary Figure S4D. After sorting for at least +/−1.5-fold change, we found that all comparisons with the HypoTH group revealed a very high number of DE mRNAs and only three genes were altered in the HyperTH vs. HypoTH + T3 group. HypoTH group vs. the Control group comparison identified 259 upregulated and 42 downregulated mRNA transcripts. HypoTH + T3 vs. HypoTH group comparison showed alteration of 115 mRNAs, of which 60 were upregulated and 55 were downregulated. For the HyperTH vs. HypoTH group, 198 mRNAs were upregulated, and 263 mRNAs were downregulated.

**mRNA Enrichment analysis:** In KEGG-enrichment analysis (Figure 6G–K), all but the HyperTH vs. Control group showed significant enrichment. All three comparisons with the HypoTH group: Control vs. HypoTH (95 pathways), HypoTH + T3 vs. HypoTH (34 pathways) and HyperTH vs. HypoTH (101 pathways) showed several significant hits. To highlight a few, their cardiovascular pathways include the TH signaling pathway, Adrenergic signaling in cardiomyocytes, Cardiac muscle contraction, DCM, HCM, ARVC, and Vascular smooth muscle contraction. Key genes of interest include *Thra*, Troponin 2 (*Tnnt2*), Myosin heavy chain 7 (*Myh7*), *Myh6*, *Pln*, *SERCA2*, *Ryr2*, Tropomyosin 3 (*Tpm3*), *NCX1*, Cardiac Myosin Binding Protein C (*Mybpc3*), etc.

All three comparisons with the HypoTH group also showed one common cancer-associated pathway: central carbon metabolism in cancer. In HyperTH vs. HypoTH and Control vs. HypoTH group comparisons, there were a few more common hits including Pathways in cancer, Proteoglycans in cancer, Endometrial cancer, and renal cell carcinoma. Control vs. HypoTH comparison also showed enrichment in Choline metabolism in cancer, Prostate cancer, and Thyroid cancer pathway genes. Although the cancer-associated pathways in mRNA KEGG analysis were not as diverse as we saw for lncRNA sequencing or miRNA sequencing, multiple common genes did emerge in these pathways including *Akt3*, *Ctnna1*, *Pdpk1*, etc. The complete KEGG Enrichment analysis is provided in Supplementary File S12.

With GO enrichment analysis, only HyperTH vs. HypoTH group had significant GO enrichment (Figure 6L) with just three GO items (one CC and two MF). The CC enrichment was Mitochondrion, and the MF hits were transferase activity, transferring acyl groups, and cytoskeletal protein binding.

#### 2.5.5. Interaction Networks

**Figure 7:**
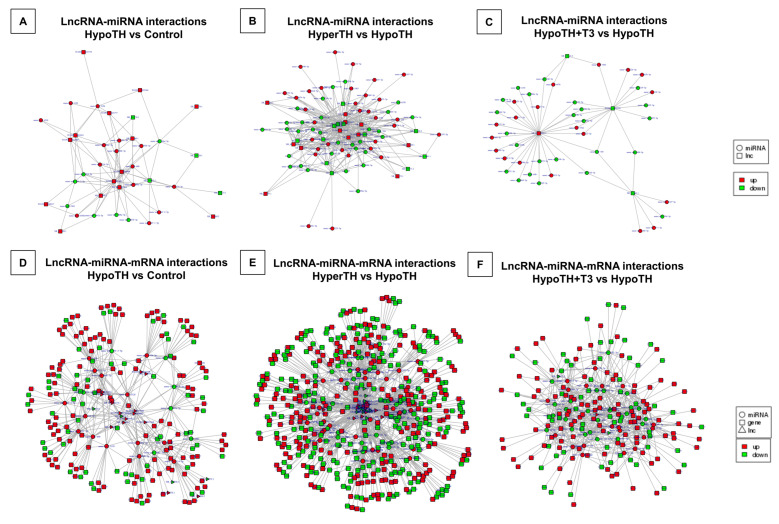
**LncRNA-miRNA and lncRNA-miRNA-mRNA correlation analyses.** Three groupwise comparisons identified the (**A**–**C**) **lncRNA-miRNA** and (**D**–**F**) **lncRNA-miRNA-mRNA** partners. (**A**,**D**) HypoTH vs. Control; (**B**,**E**) HyperTH vs. HypoTH; (**C**,**F**) HypoTH + T3 vs. HypoTH. The interaction network images were prepared in Cytoscape. Only statistically significant (*p*-adj/corr < 0.05) and differentially expressed lncRNA, miRNA and mRNA targets were included in this network analysis. Upregulated targets are marked in red and downregulated targets are marked in green.

**Correlation analysis of target lncRNA-miRNA:** To explore the cardiac lncRNA-miRNA network in altered TH states, we predicted the lncRNA targets of miRNAs using miRanda. We identified 89,399 and 56 lncRNA-miRNA potential partners in the HypoTH vs. Control, HyperTH vs. HypoTH and the HypoTH + T3 vs. HypoTH groups, respectively (Figure 7A–C). We further sorted the miRNAs that we found to be interacting with the *Thra* mRNA in the miRNA Enrichment analyses (Supplementary File S9). We thus identified multiple lncRNA partners for most of the *Thra*-interacting miRNAs, including *LOC118568312* (*TCONS_00022951*), *Gm40124*, *Cdh2*, *Gm26944*, and *LOC118568312* (*TCONS_00022949*) for *mmu-miR-29b-2-5p*; *9630028I04Rik* and *Gm52092* for *mmu-miR-708-5p*; *Synpo2*, *D330025C20Rik*, *Gm36827*, *Epb41l1*, *Sorbs2*, *LOC118568566*, *Chd9*, *Gm28979*, and *Gm10336* for *mmu-miR-511-5p*; and *D330025C20Rik*, *Foxo6os*, *Synpo2*, *Lincred1*, *Chd9*, *LOC118568566*, *E530011L22Rik*, *Gm12701*, *Gm10336*, and *Gm28979* for *mmu-miR-339-5p*. The complete interaction details and analysis for all the three groups are presented in Supplementary File S13.

***Regulatory network of lncRNA-miRNA-mRNA:*** To obtain a comprehensive view of lncRNA-miRNA-mRNA interactions, we filtered out miRNAs that target the same lncRNA and mRNA and constructed the regulatory network. We identified 6876, 49,520, and 1936 lncRNA-mRNA-miRNA interaction partners predicted for HypoTH vs. Control, HyperTH vs. HypoTH, and HypoTH + T3 vs. HypoTH groups, respectively (Figure 7D–F; *p*-adj < 0.05). Importantly, as *Thra* increases in HypoTH vs. control, the *mmu-miR-29b-2-5p* decreases along with increases in the levels of five lncRNAs, which include (i) the *XR_004934374.1* transcript of *Gm26944* lncRNA, (ii) *TCONS_00022951* transcript of *LOC118568312* lncRNA, *XR_867569.1* transcript of *Gm40124* lncRNA, *TCONS_00023682* transcript of *Cdh2* lncRNA and *TCONS_00022949* transcript of *LOC118568312* lncRNA. As the *Thra* levels are restored to control levels in HypoTH + T3 vs. HypoTH, *mmu-miR-708-5p* decreases, and (i) *TCONS_00022791* transcript of *9630028I04Rik* lncRNA decreases and (ii) *XR_003950992.1* transcript of *Gm52092* lncRNA increases. Complete lncRNA-mRNA-miRNA interaction details for all the three groups are presented in Supplementary File S14.

**Figure 8:**
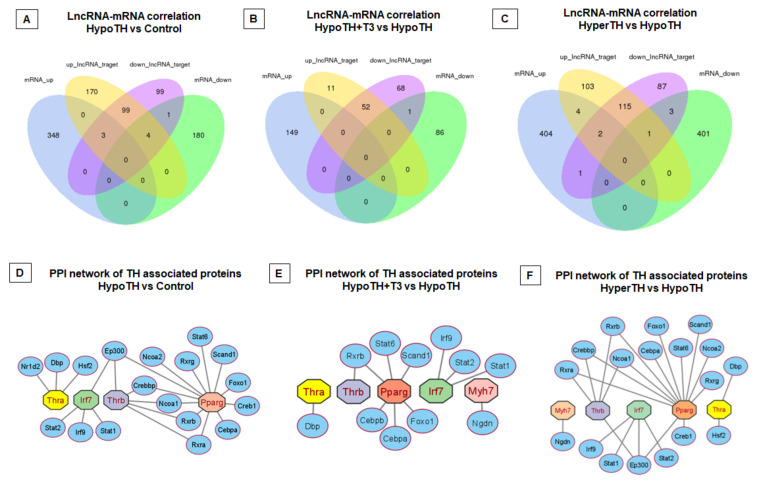
**LncRNA-mRNA correlation Analysis and PPI network. (A–C) LncRNA-mRNA correlation analysis.** Groupwise comparisons identified the LncRNA-mRNA partners in (**A**) HypoTH vs. Control, (**B**) HypoTH + T3 vs. HypoTH, and (**C**) HyperTH vs. HypoTH groups. Only statistically significant (*p*-adj < 0.05) and differentially expressed lncRNA and mRNA targets were included in this correlation analysis. (**D**–**F**) **Protein–protein interaction (PPI) network** for genes co-expressed with significantly altered lncRNAs and critically involved in cardiac or TH pathways in (**D**) HypoTH vs. Control, (**E**) HypoTH + T3 vs. HypoTH, and (**F**) HyperTH vs. HypoTH groups. The interactions that showed *p* < 0.05 were included in this analysis.

**LncRNA-mRNA Co-expression/Interaction Network:** LncRNAs can perform cis-regulatory functions by recruiting multiple chromatin remodeling factors locally [59,60,61]. These cis-regulating genes were considered coding genes that co-express with a lncRNA within about 30 kb up/downstream in the same allele. LncRNAs were also shown to trans-regulate core transcription factors to modulate their involvement in biological pathways. We evaluated potential co-expression between lncRNAs and mRNAs. A positive correlation between a lncRNA and an mRNA is assigned with a Pearson Correlation greater than 0.7 and *p* < 0.05. Statistically significant and differentially regulated lncRNA-mRNA correlation Venn diagram is shown in Figure 8A–C.

We detected 423 targets with significant cis-interactions and 60,465 targets with significant trans-interactions. Among these, we also detected lncRNA co-expression networks for major TH or cardiac signaling pathway-associated genes. These include positive or negatively correlated, cis-co-expressing lncRNAs for *Thrb*, Cardiac muscle alpha-Actin (*Actc1*), and, *Myh7*, and Tpm3. Trans-co-expression network identified the lncRNA associates for *Thra* and *Thrb*. We have identified 534 and 378 lncRNA associates trans-expressing with *Thra* and 5 transcript variants of *Thrb* (variant 1, ×1, ×14, ×18, and ×19), respectively. Moreover, we found lncRNA *Mhrt* and *Dio3os* to be trans-co-expressed with 54 and 59 mRNA transcripts. The complete list of statistically significant lncRNA-mRNA co-expression networks is provided in Supplementary Files (Cis interactions: Supplementary File S15; Trans interactions: Supplementary File S16).

**Differentially expressed lncRNA-associated protein–protein interaction (PPI) network:** The potential PPI networks were constructed using the STRING database. For this analysis, only statistically significant differentially regulated lncRNAs were taken into consideration. Accordingly, only the three aforesaid groups (HypoTH vs. Control, HypoTH + T3 vs. HypoTH, and HyperTH vs. HypoTH) provided PPIs. In addition, a minimum value of 700 was considered for the PPI Confidence score. Using Cytoscape, we further mapped the PPI networks for genes critically associated with TH and cardiac pathways (Figure 8D–F). Given the TH connection with cardiac inflammatory/immune pathways (Supplementary Figure S3) and metabolism, we investigated IRF7 and PPARg involvement in PPI. Among all the three groupwise comparisons with the HypoTH group, we have identified the PPI networks for Thra and Thrb. In HypoTH + T3 vs. HypoTH and HyperTH vs. HypoTH comparisons, we found the PPI network for Myh7. The complete results for the PPI are provided in Supplementary File S17.

**Figure 9:**
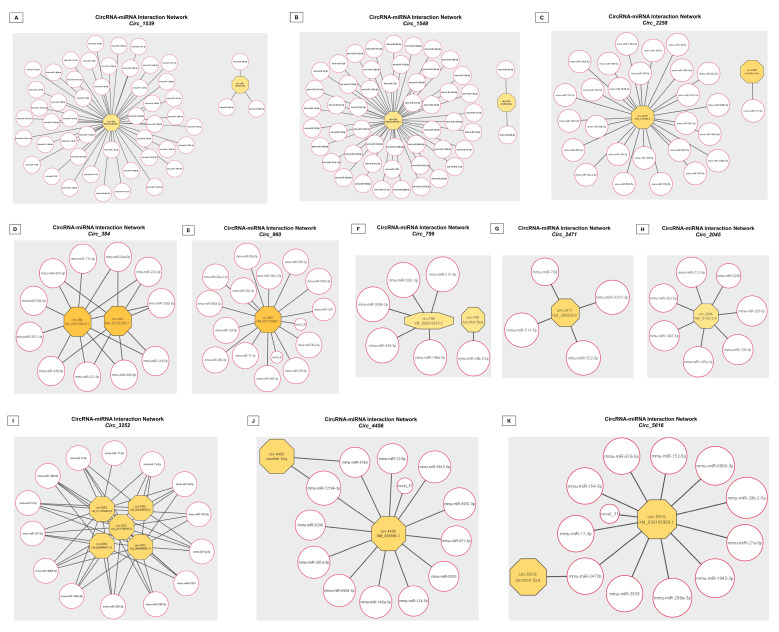
(**A**–**K**) **CircRNA-miRNA interactions for the significantly altered CircRNAs.** miRNA partners were identified and sorted for all statistically significant differentially expressed circRNAs–(**A**) *Circ_1539*, (**B**) *Circ_1548*, (**C**) *Circ_2258*, (**D**) *Circ_384*, (**E**) *Circ_960*, (**F**) *Circ_799*, (**G**) *Circ_2471*, (**H**) *Circ_2045*, (**I**) *Circ_3252*, (**J**) *Circ_4456*, and (**K**) *Circ_5616*. Network images were prepared with Cytoscape. *p* < 0.05 were considered statistically significant.

**Prediction of circRNA-miRNA binding partners:** Following the identification of significantly altered circRNAs in response to changes in physiological TH levels, we also constructed the miRNA interaction network for each of the 11 circRNAs. A total of 1033 miRNA interactions were predicted for the DE circRNAs. Among them, 10 novel and 176 annotated unique miRNA-interacting partners were identified for the select 11 circRNAs. Representative 50 binding interactions for each of these DE circRNAs were prepared using Cytoscape (version 3.9.1; Figure 9A–K). The complete list of circRNA-miRNA interactions for 11 DE circRNAs is provided in Supplementary File S18.

**miRNA and mRNA correlation analysis–*miRNA-mRNA enrichment analyses:*** Three comparisons showed significant KEGG pathway enrichments: Control vs. HypoTH (118), HypoTH + T3 vs. HypoTH (58), and HyperTH vs. HypoTH (118). Among several other pathways, significantly enriched cardiac mechanisms include Adrenergic signaling in cardiomyocytes, ARVC, DCM, HCM and Cardiac muscle contraction. Many cancer-associated pathways also emerged in our analysis including miRNAs in cancer, pathways in cancer, thyroid cancer, endometrial cancer, prostate cancer, colorectal cancer, Central carbon metabolism in cancer, proteoglycans in cancer, choline metabolism in cancer, transcriptional misregulation in cancer, pancreatic cancer, and small cell and non-small cell lung cancers. Complete KEGG analysis results are provided in Supplementary File S19. For GO enrichment analysis, only the HyperTH vs. HypoTH comparison had only one significantly enriched GO component—MF, which particularly included several nucleoside and nucleotide binding pathways. We found no other statistically significant GO enrichment in the other comparisons.

**Figure 10:**
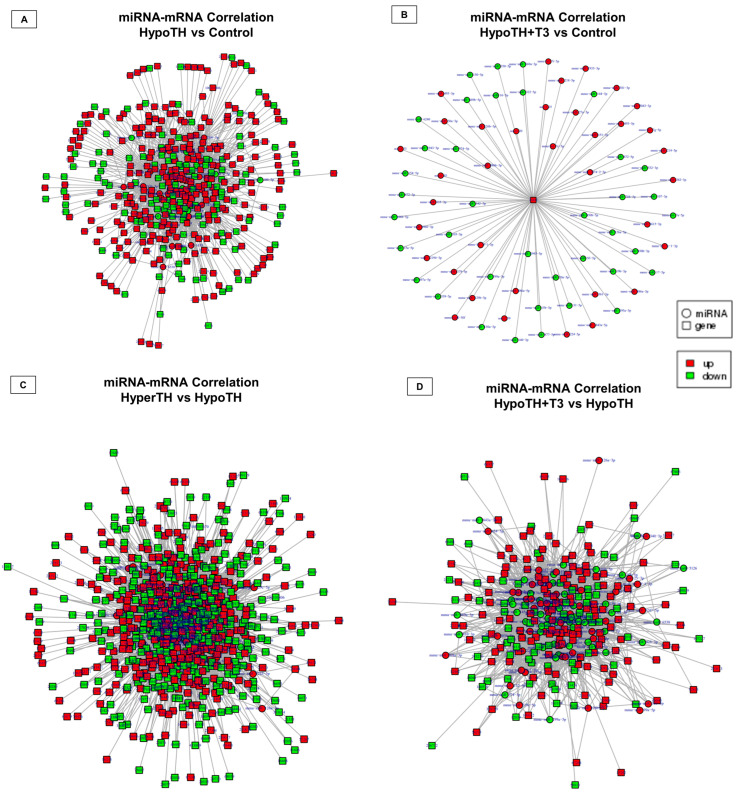
**miRNA-mRNA correlation analysis.** Groupwise comparisons identified the miRNA-mRNApartners in (**A**) HypoTH vs. Control, (**B**) HypoTH + T3 vs. Control, (**C**) HyperTH vs. HypoTH, (**D**) HypoTH + T3 vs. HypoTH groups. The interaction network images were prepared in Cytoscape. Only statistically significant (*p*-adj/corr < 0.05) and differentially expressed miRNA and mRNA targets were included in this network analysis. Upregulated targets are marked in red and downregulated targets are marked in green.

**miRNA and mRNA correlation analysis–*miRNA-mRNA network analysis:*** We analyzed and sorted miRNA-interaction partners for DE mRNAs shown in Figure 6. Except for the HypoTH + T3 vs. HyperTH group and HyperTH vs. Control group, we have identified miRNA partners for a significant portion of selected mRNAs for all other groups (Figure 10A–D). We have identified a total of 1524, 6276, 2508 and 74 mRNA-miRNA interactions in HypoTH vs. Control, HyperTH vs. HypoTH, HypoTH + T3 vs. HypoTH, and HypoTH + T3 vs. Control groups, respectively. In the HypoTH + T3 vs. Control group, all the 74 miRNAs were statistically significantly found to interact only with *Skil.* This gene is known to play a key role in response to extracellular growth signals and regulating members of the transforming growth factor-beta signaling pathway. Key genes in the cardiac–thyroid pathway included *Thra*, *Mybpc3*, *Ryr2*, Myosin VIIA (*Myo7a*), and *NCX1*. *Thra*-interacting miRNAs included *miR-29b-2-5p* in HypoTH vs. control group, and *miR-106b-5p*, *miR-511-5p*, *miR-378a-5p*, *miR-339-5p*, and *miR-708-5p* in HypoTH + T3 vs. HypoTH group. Within the HyperTH vs. HypoTH comparison, we have identified *mmu-miR-339-5p* as an interacting partner of *Ryr2*. Complete interaction details for all the groupwise comparisons are available in Supplementary File S20.

**Differentially expressed mRNA-associated PPI network:** The potential PPI networks for DE mRNA transcripts were constructed using the STRING database. Out of the five comparisons that showed significantly altered mRNAs, only the three comparisons with HypoTH showed PPI with at least the cut-off PPI confidence score of 700. We have identified 873, 145, and 1352 interactions in comparisons of the HypoTH group with Control, HypoTH + T3, and HyperTH groups, respectively. The complete DE mRNA-associated PPI results are provided in Supplementary File S21.

## 3. Discussion

Non-coding RNAs contribute to the majority of the genome with vast parts being actively transcribed. They are now increasingly recognized as critical mediators and regulators in numerous cardiovascular physiological and pathological processes [4,62]. In this study, we have unraveled novel cardiac transcriptomics both when TH levels are abnormal and when they are restored to normal levels following T3 treatment. The restoration of serum T3 to control levels, without creating a state of hyperthyroidism, also correlated well with the tissue morphometrics. Thus, this also serves as an excellent approach for studying TH-mediated effects on the heart (both the ventricles) and also on the kidneys (both right and left). A mouse model of right ventricular hypertrophy and dysfunction following pulmonary artery constriction reported that downregulated pairs of coding/non-coding RNAs were enriched in the TH signaling pathway in cardiac tissues [63]. However, this is the first study to present a comprehensive outlook of LV cardiac noncoding RNAs and their interactions under altered TH states.

### 3.1. All Major Cardiac Noncoding RNAs Are Regulated by Systemic TH Levels

We and others have shown that serum HypoTH state can induce or be associated with HypoTH in the cardiac tissues as well (rodents and humans) [27,40,64,65,66]. We recently showed that *MALAT1* lncRNA expression was diminished in the LVs following T3 administration in a model of acute caloric restriction [52]. Another study showed T3-dependent regulation of *Myh* switch via activation of *Mhrt* lncRNA promoter at two putative TH-responsive elements via *miR-208a* [67]. In our current study, *Synpo2* and *mt-Rnr2* lncRNAs were significantly upregulated in HypoTH and downregulated in HyperTH. *Gm42067* was significantly downregulated in HypoTH and upregulated in HyperTH (Figure 3 and Supplementary Tables S2–S4). The *Fam120aos* lncRNA was found to be significantly downregulated in response to both T3 (2.21-fold) and T4 (HyperTH; 2.50-fold). Importantly, the increase in *Gm52092* expression levels in HypoTH + T3 was 1.5 times lower than that of HyperTH. These indicate that the regulation of these lncRNAs in the heart potentially via local cardiac tissue TH signaling and are worthy of further investigation.

KEGG analysis of plasma samples from patients after isolated off-pump coronary artery bypass grafting indicated that *hsa_circ_025016* is predicted to participate in the TH signaling pathway [68]. Our current circRNA analyses revealed 11 circRNAs that were downregulated in response to HyperTH stimulation, with *mmu_circ_1548* being the most significant of all (Supplementary Table S5). To our knowledge, no reports have been shown on these targets and warrant further investigation. Numerous studies have shown the vital roles of miRNAs in cardiovascular disorders [16,17,18,48,49,50,51]. In this study, we have also uncovered hundreds of significantly altered and novel miRNAs in TH-mediated cardiac regulation (Supplementary File S7). Notably, one of the miRNA enrichment pathways includes brain natriuretic peptide (BNP; *Nppb*) in the multiple comparisons. This reinforces the importance of BNP as a biomarker of cardiac TH physiology and pathophysiology by tracking cardiac tissue T3 levels [45,69], which will be significantly altered in all the groups in this study [66].

In the mRNA sequencing, within the HypoTH + T3 and HyperTH group comparison, two mitochondria-related genes showed significant alterations. These include *Supv3l1* being 6.36-fold downregulated and *Mrpl14* being 22.37-fold upregulated in the HyperTH group. Both these genes are involved in mitochondrial RNA or protein regulation processes. The other gene that was significantly upregulated in the HyperTH group compared to HypoTH + T3 was methyltransferase *Mettl9*. *Mettl9*-mediated methylation is known to enhance respiration via mitochondrial electron transport chain complex 1 [70]. It will be interesting to further explore the mechanistic basis underlying dysregulation of these mitochondrial pathway-associated genes in pathological HyperTH state versus the systemic restoration of euthyroid state (HypoTH + T3) [50,71,72,73].

In the enrichment analyses, the four significant cardiac pathways that emerged were Cardiac muscle contraction, DCM, ARVC, and HCM. Among the genes or host genes, a few important ones were Troponin T (*TnnT*), *Myh7*, and *Myh6* [38,74,75]. Particularly in the circRNA enrichment analysis, Titin (*Ttn*) and cardiac *Ryr2* were identified as two of the host genes associated with HCM and DCM pathways. In addition, *Ryr2* was also found to be the host gene associated with ARVC and cardiac muscle contraction-related pathways.

### 3.2. Novel Interaction Networks of Cardiac Non-Coding and Coding RNAs

We uncovered numerous interacting partners for lncRNA-miRNA, lncRNA-mRNA (cis and trans), lncRNA-miRNA-mRNA, circRNA-miRNA, miRNA-mRNA, and mRNA-PPI networks. In addition, we also identified several thousands of significant interactions among lncRNAs, miRNAs, and mRNAs. These act in concert with one another to exert major physiological and pathological effects [4,8,54,55,56]. For instance, we discovered a novel pathway by which *Thra* may play its crucial role in the mammalian heart. As *Thra* increases in HypoTH, the *mmu-miR-29b-2-5p* decreases, and levels of five lncRNAs increase. These lncRNAs include the (i) *XR_004934374.1* transcript of *Gm26944* lncRNA, (ii) *TCONS_00022951* transcript of *LOC118568312* lncRNA, (iii) *XR_867569.1* transcript of *Gm40124* lncRNA, (iv) *TCONS_00023682* transcript of *Cdh2* lncRNA, and (v) *TCONS_00022949* transcript of *LOC118568312* lncRNA. As the *Thra* levels are restored to control levels in HypoTH + T3 (vs. HypoTH), *mmu-miR-708-5p* decreases, and (i) *TCONS_00022791* transcript of *9630028I04Rik* lncRNA decreases and (ii) *XR_003950992.1* transcript of *Gm52092* lncRNA increases (Figure 7D–F and Supplementary File S14).

In addition to the pathway targets, we have also identified the PPI partners for key TH, cardiac, and inflammation pathway-associated genes, including *Thra*, *Thrb*, *Myh7*, *Pparg,* and *Irf7*. For instance, with HypoTH, *Thra* was interacting with *Nr1d2*, *Dbp*, and *Hsf2*, whereas following T3 restoration, *Thra* was interacting with *Dbp*. Understanding these novel mechanisms may offer better therapeutic options for tissue repair [76].

### 3.3. Non-Coding RNAs in Cardio-Thyro-Oncology

A closer look into our findings revealed associations of altered TH states with DE of genes in several cancer-associated pathways. Based on the GO and KEGG enrichment analyses, these include thyroid cancer, acute myeloid leukemia, chronic myeloid leukemia, prostate cancer, pancreatic cancer, small cell and non-small cell lung cancers, prostate cancer, endometrial cancer, renal cell carcinoma, colorectal cancer, central carbon metabolism in cancer, pathways in cancer, proteoglycans in cancer, choline metabolism in cancer, viral carcinogenesis, and pathways involved in transcriptional misregulation in cancer. In the CircRNA enrichment analysis, among the cancer-associated pathways, only *Nfkb1*, was found to be involved in all types of cancer and cancer-associated pathways. *Nfkb1* was also associated with the highest number of enriched pathways. In addition, our model may also serve as a tool to investigate cardiac atrophy (and its rescue) that can also be seen in cancer [77].

Clinical studies have reported alteration in TH levels [46,47] as well as shrinkage in thyroid gland size [78] in patients undergoing or following chemotherapy. Moreover, THs can enhance the efficacy of chemotherapy [79]. Furthermore, cardiac metastases of anaplastic thyroid carcinoma have also been reported [80]. It will be useful to conduct detailed explorations into how alterations in systemic and local tissue TH levels could affect the heart in patients with various types of cancer with or without chemotherapy (that may cause cardiotoxicity). This might also lead to the development of novel adjuvant therapy avenues. These findings may also offer the potential to develop a sub-field in Cardiooncology, which may be termed *cardio-thyro-oncology*.

## 4. Materials and Methods

### 4.1. Animal Models

**Figure 11:**
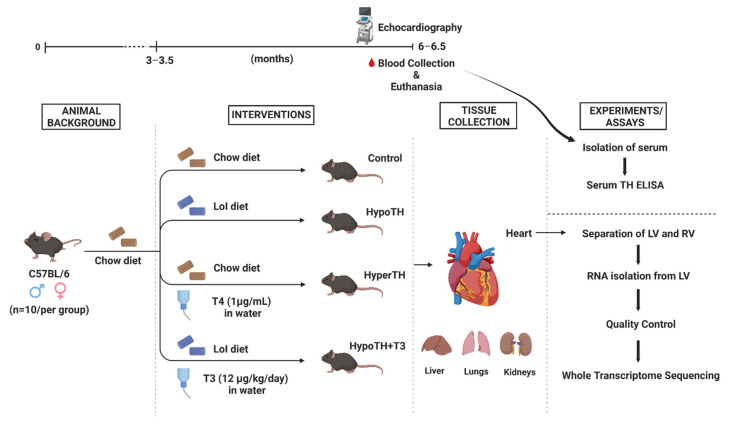
**Experimental protocol design leading to WTS:** Complete protocol and timeline is presented for the animal experiments in this study (the image was created with biorender.com). LoI: Low iodine; HypoTH: Hypothyroidism; HyperTH: Hyperthyroidism; TH: Thyroid hormone; T3: Triiodothyronine; T4: Thyroxine; LV—Left Ventricle; RV—Right Ventricle; ELISA—Enzyme-linked immune sorbent assay; WTS—Whole Transcriptome Sequencing.

All protocols were approved by the Institutional Animal Care and Use Committee at Arkansas State University and were performed in accordance with the Guidelines for the care and use of laboratory animals. Animals were housed in the Arkansas Biosciences Institute Animal Care Facility and maintained on a 12:12 h light-dark cycle. A flowchart of animal groups, treatment interventions, number of animals, timeline, etc. is provided in Figure 11. Adult 3–3.5 months old, wild-type C57BL/6 (Jax), mixed-sex (8–10 per group; with 3–5 males and females each) mice were randomly separated into placebo, hypothyroid (HypoTH), hyperthyroid (HyperTH), and T3 treatment following HypoTH induction (HypoTH + T3) groups. HypoTH group mice received a low iodine diet (Envigo, Indianapolis, IN, USA) and water containing 0.1% *w*/*v* methimazole (Sigma, St. Louis, MO, USA), 1% *w*/*v* along with sodium perchlorate (Sigma, St. Louis, MO, USA) and 3 g/L saccharin (Sigma, St. Louis, MO, USA) [81]. Due to early mortality, the methimazole treatment was discontinued. The HyperTH group received a standard chow diet along with 1 µg/mL T4 (Sigma, St. Louis, MO, USA) in 40 mM NaOH and 0.1% bovine serum albumin (BSA) in drinking water [82]. Another group that received the HypoTH diet also received water containing T3 (12 ug/kg/day) with 0.04 M NaOH and 0.02% BSA. Due to the light sensitivity of T3 and T4, the stocks and treated water were kept away from light at all times. Compounds in water were prepared fresh and changed twice a week. The control group mice were fed the standard chow diet and received diluents at the same volume and frequency. All groups received the aforementioned diets or treatments for about 3 months until euthanasia.

### 4.2. Echocardiography

Echocardiographic measurements were collected via a SonoSite M-Turbo ultrasound system coupled with an ultrasound transducer probe. Following isoflurane induction, mice were anesthetized with 1.5% isoflurane for maintenance across all groups. Subsequently, the chest hairs were shaved, and the mice were secured warm on an isothermal pad at 37 °C throughout the imaging procedure. Two-dimensional (2D) echocardiograms were obtained from short-axis views of the LV to measure the physiological parameters in systole and diastole.

### 4.3. Tissue Isolation and Sample Procurement

Immediately following isoflurane overdose euthanasia and opening of the chest, blood was collected from the apex of beating LV. Subsequently, diastolic cardiac arrest was induced with 20 mM KCl. The hearts were immediately dissected out to cold buffer, blood was removed, and LV and RV were separated out. In addition to the ventricles, kidneys, liver, and lungs were all quickly weighed before they were flash-frozen and transported in liquid N_2_. The collected tissues were saved at −80 °C until further use.

### 4.4. Serum Thyroid Hormone Levels

The collected blood was separated into serum by centrifugation at 4000 RPM for 15 min at 4 °C. Serum samples were aliquoted and saved at −80 °C until use. Serum TH levels were assessed in a blinded manner using total T3 and total T4 enzyme-linked immunosorbent assay (ELISA) kits (Monobind Inc., Lake Forest, CA, USA), according to the manufacturer’s instructions.

### 4.5. Tissue Processing and RNA Isolation

Frozen LV tissues were minced cold if needed and transferred into RNase-free Rino lysis kit tubes (Next Advance, Troy, NY, USA) with Trizol reagent (Invitrogen, Waltham, MA, USA). The tissues were homogenized at 4 °C in Bullet Blender (Next Advance, Troy, NY, USA) until no chunks of tissues were visible. Following incubation, chloroform was mixed to the homogenized tissues and subjected to centrifugation at 12,000× *g* for 30 min at 4 °C. The upper aqueous layer was then carefully separated in to RNAse-free tubes, and an equal volume of RNAse-free 70% ethanol was added to it. The mixture was then transferred on to Purelink RNA mini kit (Invitrogen, Waltham, MA, USA) binding columns and processed based on a slightly modified manufacturer’s protocol. Additional genomic DNA removal step was performed using RNase-free DNase (Qiagen, Germantown, MD, USA). The concentration and quality of the isolated total RNA were initially verified with Nanodrop (Thermo Fisher Scientific, Waltham, MA, USA).

### 4.6. Quantitative Real-Time PCR

For the quantitative real-time PCR (qRT-PCR) array, a separate group of female mice were used (*n* = 3–4/group) with control and HypoTH drug/treatment protocol as mentioned earlier but for 5 weeks. The HyperTH group received a standard chow diet along with intraperitoneal injections of T4 (1 µg/g in 0.01 M NaOH, 0.1% BSA) every other day. The control group mice were fed a standard chow diet and received intraperitoneal injections of phosphate-buffered saline every other day at the same volume as the HyperTH group. The isolated RNA was converted to cDNA using RT^2^ first-strand synthesis kit (Qiagen, Germantown, MD, USA). qPCR was performed using RT^2^ lncRNA PCR array (Qiagen, Germantown, MD, USA) and RT^2^ SYBR Green qPCR Mastermix (Qiagen, Germantown, MD, USA) in CFX384 (BioRad, Hercules, CA, USA). Normalization was performed with *Rplp0* (Ribosomal protein, large, P0). Automated data analysis was performed with Qiagen GeneGlobe using the 2^−ΔΔCT^ method. Fold change of at least ±1.5 and *p* < 0.05 was considered significant. A total of 86 distinct lncRNAs associated with inflammatory/autoimmune pathways were studied with appropriate controls following pre-amplification. All the samples passed for quality control checks including array reproducibility, reverse transcription efficiency and genomic DNA contamination.

### 4.7. RNA Library Preparation and Whole Transcriptome Sequencing

Whole transcriptome RNA-seq was conducted in a blinded manner to investigate lncRNAs, circRNAs, miRNAs, and mRNAs. Total RNA was shipped to Novogene (Beijing, China) under frozen conditions throughout.

### 4.8. Sample Quality Control

The sample quality was validated by (i) Nanodrop (ii) Agarose Gel Electrophoresis (iii) Agilent Bioanalyzer 2100 system with High Sensitivity Chips.

The following methodologies were common for all the RNA species sequenced in this study. Methodologies specific to any type of RNA are discussed separately.

LncRNA and circRNA libraries were prepared with a maximum of 5 µg of total RNA. The ribosomal RNA was removed from the samples by Epicentre Ribozero rRNA removal kit (Epicentre, Madison, WI, USA) followed by a cleanup by ethanol precipitation. The linear RNA was digested with 3 U of RNaseR (Epicentre, Madison, WI, USA) and the sequencing libraries were generated by NEBNext Ultra Directional RNA Library Prep kit for Illumina (NEB, Ipswich, MA, USA) according to the manufacturer’s protocol.

A maximum of 3 µg RNA was used as input material for small RNA library preparation. NEBNext Multiplex Small RNA Library Prep Set for Illumina (NEB, Ipswich, MA, USA) was used following the manufacturer’s protocol to generate sequencing libraries and to attribute sequences to every sample, index codes were added. The library quality was verified on the Agilent Bioanalyzer 2100 system using High Sensitivity Chips.

### 4.9. Data Quality Control

FASTQ format raw data were processed by Novogene in-house scripts. From the raw data, reads containing adapter, poly-N sequences and low quality were removed and Q20, Q30 and GC contents of the clean data were calculated. Only clean data of high quality were used for all the downstream analyses.

To identify novel transcripts, first, the set of transcripts obtained in the mapping step was assembled using StringTie [83,84]. Cuffcompare was then used to compare the StringTie assemblies to reference annotation files and sort new transcripts from the known ones [85]. rMATS software was used for alternative splicing analysis [86]. Expanded methodology for library preparation and sequencing can be found in the Supplementary Materials (Methods and Results File).

### 4.10. Statistical Analyses

All data in this article are expressed as mean ± standard error, unless noted otherwise (e.g., standard deviation). Statistical data analyses were performed with GraphPad Prism (Version 9.4.0) software. Analyses of groupwise comparisons were carried out a using two-tailed student’s *t*-test or one-way analysis of variance (ANOVA) with a Tukey’s multiple comparisons test, and a *p*-value < 0.05 was considered statistically significant. Analyses for sequencing were performed by Novogene Bioinformatics (Novogene, Sacramento, CA, USA).

### 4.11. LncRNA Data Analyses

The mapped reads of every sample were first assembled by StringTie. These transcripts were analyzed for their protein-coding potential to identify novel transcribed regions using three analytical tools: CNCI (Coding-Non-Coding Index) [87], CPC (Coding potential Calculator) [88], and Pfam [89]. Potential cis and trans co-expression between lncRNA and mRNAs were evaluated using Pearson Correlation analysis. Significantly positive correlation had a value greater than 0.7 and the *p*-value was less than 0.05. For DE analysis, Cuffdiff was used and transcripts with *p*-adjust < 0.05 were marked as DE between any two groups. GOseq R package [90] was used for Gene ontology (GO) analysis after correcting the gene length bias. The GO terms with a corrected *p*-value < 0.05 were described as significantly enriched. For KEGG [91] pathway enrichment analysis, KOBAS [92] was used for testing statistically enriched DE lncRNA targets. Expanded methodological details can be found in the Supplementary Materials (Methods and Results File).

### 4.12. CircRNA Data Analyses

A similar approach was used as lncRNAs above. CircRNAs were identified using CIRI2 [93] and find_circ [94]. Prior to differential gene expression analysis, the read counts were adjusted by edgeR for each sequenced library through one scaling normalized factor. Then, DE analysis was performed with the edgeR R. The *p* values were adjusted using the Benjamini and Hochberg methods with a corrected *p*-value of 0.05 set as the threshold for significant DE. GOseq R was used for gene ontology (GO) enrichment analysis for host genes of DE circRNA and the gene length bias was corrected. GO terms with corrected *p*-value < 0.05 were considered significantly enriched by DE genes. To identify and test the statistical enrichment of circRNA host genes in the KEGG pathways, KOBAS was used. Furthermore, miRanda was used to identify miRNA target sites within the exons of circRNAs, and the network images were constructed using Cytoscape [95]. Additional methodological details can be found in the Supplementary Materials (Methods and Results File).

### 4.13. Small RNA Data Analyses

For miRNAs, the index-coded samples were clustered on a cBot Cluster Generation System using TruSeq SR Cluster Kit v3-cBot-HS (Illumina, San Diego, CA, USA) following the manufacturer’s protocol. After clustering, the library preparation was sequenced on an Illumina platform, generating 50 bp single-end reads. To analyze the expression and distribution of small RNAs on the reference, the small RNA tags were mapped to the reference sequence using Bowtie [96] without mismatch. The tags originating from protein-coding genes, repeat sequences, rRNA, tRNA, snRNA, and snoRNA were mapped to RepeatMasker, Rfam database to sort out small RNAs. Next, to identify the known small RNAs, mapped small RNA tags were used. miRBase20.0 was used as the reference, and a modified version of mirdeep2 software [97] and srna-tools-cli were used to identify potential miRNAs. miREvo [98] and mirdeep2 [97] software were integrated to predict novel miRNAs. As the characteristics of the hairpin structure of miRNA precursors can be utilized to predict novel miRNAs, multiple parameters were integrated including secondary structure, Dicer cleavage site, and minimum free energy of the small RNA tags unannotated during previous steps. miRNA expression levels were estimated by TPM (transcript per million) [99]. DE analysis was performed using DEGseq [100] R package (1.8.3). qvalue [101] <0.05 was set as the threshold for significant DE. GO enrichment analysis was used on the target gene candidates of DE miRNAs. GOseq-based Wallenius non-central hyper-geometric distribution [90], which could adjust for gene length bias, was implemented for GO enrichment analysis. For KEGG pathways [91], KOBAS [92] was used to test the statistical enrichment of the target gene candidates. Expanded methodological details can be found in the Supplementary Materials (Methods and Results File).

### 4.14. Correlation and Interaction Analyses

For all groupwise comparisons, the DE genes were identified and functionally annotated, and the miRNA candidates were identified, which are possibly involved in regulating these mRNAs. Similarly, the aforementioned protocol for miRNA candidates was performed in all the groupwise comparisons using miRanda. The candidate target miRNAs for each DE mRNA and further enrichment analyses (GO and KEGG) were performed for (a) down-regulated miRNA and upregulated mRNA and (b) up-regulated miRNA and down-regulated mRNA for all the groups. As above, the GOseq R package [90], was used for GO enrichment analysis. A similar methodology was employed to identify the lncRNA targets of miRNAs. For lncRNA-miRNA correlation analysis, first, the lncRNAs that can potentially be the precursors of miRNAs were excluded by studying the homology of the lncRNA and miRNA precursors. Then, the target lncRNAs for the miRNAs were identified with miRanda. Similarly, circRNA-miRNA binding partners were also identified using miRanda. For constructing the circRNA-miRNA interaction networks, miRNA partners for each of the 11 differentially regulated circRNAs were sorted and used as the input for network construction in Cytoscape. In addition, the lncRNAs and mRNAs targeted by the same miRNAs were filtered out, and the lncRNA-miRNA-mRNA networks were constructed. The protein–protein interaction (PPI) networks were constructed using the STRING database and further mapped using Cytoscape.

## 5. Limitations

Despite the fact that some biological features differ between animals and humans, about 99% of the mouse protein-coding genes are considered to have a homolog in the human genome [102,103]. In addition, about 90% of the human and mouse genome regions have comparable synteny. Although the animals used for the WTS were only males (due to constraints in tissue volume requirements), we have shown in this study that the physiological effects were largely similar across both the sexes following all the three interventions. In addition, our real-time PCR assay was performed in female mice. Tibial length data are not available to normalize with changes in organ weights. Since the HypoTH group had smaller hearts, total RNA from one of the apical LVs was insufficient to ensure quality control. Therefore, for lncRNAs and circRNAs, a mix of upper and lower LVs was used, and for small RNA, upper LV was used for this sample. All other samples from all the groups, including from the rest of the HypoTH, were from the lower apical part of the LVs.

## 6. Conclusions

This is the first comprehensive and comparative analysis of the whole cardiac transcriptome and interactome of modified and clinically relevant TH states. The findings offer an abundant repository of significant big data for valuable future investigations at molecular, structural, functional, and clinical levels in essentially all possible pathways across the length and breadth of cardiac omics.

## Figures and Tables

**Table 1:** Echocardiogram shows preserved cardiac function following TH treatment.

	Control	HypoTH	HyperTH	HypoTH + T3
**Heart rate (bpm)**	464 ± 32.7	337 ± 32.4 ****	510 ± 27.6 ^####^	515 ± 58.7 ^####^
**FS (%)**	42.6 ± 6.6	28.2 ± 6 ***	43.7 ± 5.4 ^####^	48.5 ± 5.6 ^####^
**IVSd (mm)**	0.82 ± 0.2	0.56 ± 0.1	0.86 ± 0.2 ^#^	0.99 ± 0.2 ^###^
**IVSs (mm)**	1.36 ± 0.2	1.02 ± 0.1 **	1.59 ± 0.1 *^,####^	1.75 ± 0.2 ***^,####^
**LVIDd (mm)**	3.53 ± 0.2	3.59 ± 0.5	4.27 ± 0.5 *^,#^	3.78 ± 0.5
**LVIDs (mm)**	2.02 ± 0.2	2.59 ± 0.5	2.41 ± 0.4	1.96 ± 0.4 ^#^
**LVPWd (mm)**	0.82 ± 0.3	0.58 ± 0.2	0.87 ± 0.1	0.87 ± 0.4
**LVPWs (mm)**	1.16 ± 0.5	0.71 ± 0.2 ^€^	1.28 ± 0.2 ^##^	1.25 ± 0.4 ^#^

HyperTH: Hyperthyroidism; HypoTH: Hypothyroidism; T3: Triiodothyronine; IVSd and IVSs: Interventricular septal in diastole and systole, respectively; LVIDd and LVIDs: Left ventricular internal diameter end diastole and end systole, respectively; FS: % Fractional Shortening; LVPWd and LVPWs: Left ventricular posterior wall diameter in diastole and systole, respectively; *n* = 6–9/group; Means ± standard deviation; * *p* < 0.05; ** *p* < 0.01; *** *p* < 0.001; **** *p* < 0.0001 vs. Control; ^€^ *p* = 0.06 vs. Control; ^#^ *p*< 0.05; ^##^ *p* < 0.01; ^###^ *p* < 0.001; ^####^ *p* < 0.0001 vs. HypoTH group.

## Data Availability

Data will be made available in public datasets.

## Supplementary Materials

The following supporting information can be downloaded at: https://www.mdpi.com/article/10.3390/ijms24076560/s1.
