# Supplementary material for: Novel Transcriptomic Interactomes of Noncoding RNAs in the Heart under Altered Thyroid Hormonal States
Source: Int J Mol Sci. 2023 Mar 31;24(7):6560. doi: 10.3390/ijms24076560 (PMC10095585; doi:10.3390/ijms24076560)
Supplement: Supplementary file 1 [file ijms-24-06560-s001.zip › Supplementary Methods and Results.pdf]

SUPPLEMENTAL METHODS AND RESULTS

SUPPLEMENTAL MATERIALS:

LncRNA:

LncRNA Preparation and sequencing:
The workflow is as follows: Total RNA sample RNA test → rRNA removal → double-stranded cDNA synthesis → repair ends, add "A" overhang and adaptor → cDNA second-strand degradation → fragment selection and PCR amplification → library quality test → Illumina sequencing.

Mapping to reference genome:
Reference genome and gene model annotation files were downloaded from a genome website browser (NCBI/UCSC/Ensembl) and paired-end clean reads were mapped to the reference genome using HISAT2 software.

LncRNA and mRNA identification and Quantification:
Mapped read assembly was performed by StringTie. StringTie, with '--library-type' and other default parameters, was used to assemble the mapped reads of each sample. All the transcripts from all the samples were merged through Cuffmerge. StringTie-eB was used to obtain an estimate of both lncRNAs and mRNA transcripts in each sample. By summing up the transcript fragments per kilo-base of exon per million fragments mapped (FPKMs) in each gene group, gene FPKMs were computed.

Novel gene prediction:
StringTie (genome-guided transcriptome assembly approach) was used to assemble the set of transcripts obtained during the mapping step and Cuffcompare was used to compare StringTie assemblies to reference annotation files and help sort out new genes from known ones. The class codes are as follows: "u" for "Potentially novel gene" and the set of "j" (Potentially novel isoform: at least one splice junction is shared with a reference transcript), "u" (Unknown, intergenic transcript), "i" (A transfrag falling entirely within a reference intron), "o" (Generic exonic overlap with a reference transcript), and "x" (Exonic overlap with reference on the opposite strand) for "Potentially novel isoform".

LncRNA Target prediction:
Potential co-expression between lncRNAs and mRNAs was evaluated using Pearson Correlation. A correlation >0.7 and a p-value<0.05 was considered positive.

Alternative splicing analysis:
rMATS was utilized to perform alternative splicing analysis. Alternative splicing events for all major types of alternative splicing patterns were identified and the *P* value and FDR were calculated for differential splicing. These include exon skipping (SE), alternative 5′ splice sites (A5SS), alternative 3′ splice sites (A3SS), mutually exclusive exons (MXE), and retained introns (RI).

Variant Calling:
Picard tools and Samtools were used to sort, mark duplicated reads and reorder the bam alignment results of each sample, followed by HaplotypeCaller tool in GATK software to complete variant discovery, including SNPs and INDELs. Finally, ANNOVAR was used to functionally annotate genetic variants detected from diverse genomes.

CircRNA:

CircRNA Preparation and sequencing:
The workflow is as follows: Total RNA QC → rRNA digestion → linear RNA digestion → dsDNA synthesis → adapter ligation → size selection and cDNA second-strand digestion → PCR library QC and sequencing.

Quality control and mapping to reference genome:
FASTQ format raw data were processed by Novogene in-house scripts. The reference genome and gene model annotation files were downloaded from genome website browser (NCBI/UCSC/Ensembl) and Paired-end clean reads were mapped to the reference genome using Spliced Transcripts Alignment to a Reference (STAR) software, which exhibits better alignment precision and sensitivity than other RNA-seq aligners for both experimental and simulated data.

CircRNA identification and distribution:
CIRCexplorer2 was used to detect circRNAs as well as to identify alternative back-splicing and back-spliced circRNA variants. Step-1 includes Step1: Circular RNA fusion junction read alignment and parsing; Step2: De novo assembly for circular RNA transcripts; Step3: Characterization of alternative back-splicing, including two types of alternative back-splicing events (alternative 5' back-splice site and alternative 3' back-splice site). The density of total mapped reads in each chromosome was calculated. Circos was used to analyze the relationship between chromosome length and total reads.

Quantification:
The expression of circRNAs is represented by the fragments that are mapped to the back-spliced exon–exon junction sites. Normalized RNA-seq fragments that are mapped to a specific back-spliced exon–exon junction by total mapped fragments were used along with the raw fragment numbers to quantitate circRNA expression. With FPM (Fragments mapped to back-spliced junction Per Million mapped fragments), circRNAs from different samples with distinct sequencing depths were compared. FPM = (Fragments mapped to a specific back-spliced junction x 10^6)/Total mapped fragments

Small RNA:

Library preparation for Small RNA sequencing:
From total RNA, sequencing libraries were generated using NEBNext® Multiplex Small RNA Library Prep Set for Illumina® (NEB, USA) and index codes were added to attribute sequences to each sample. Briefly, NEB 3' SR Adaptor was directly and specifically ligated to the 3' end of miRNA, siRNA and piRNA. The SR RT Primer hybridized to the excess of 3' SR Adaptor and transformed the single-stranded DNA adaptor into a double-stranded DNA molecule. The 5′ends adapter was ligated to 5′ends of miRNAs, siRNA and piRNA. The first strand cDNA was synthesized using M-MuLV Reverse Transcriptase (RNase H^{-}). PCR amplification was performed, and PCR products were purified on an 8% polyacrylamide gel. DNA fragments corresponding to 140~160 bp (the length of small noncoding RNA plus the 3' and 5' adaptors) were recovered and dissolved in elution buffer. The library quality was assessed on the Agilent Bioanalyzer 2100 system using High Sensitivity Chips.

Quality control:
FASTQ format raw data were processed by Novogene in-house perl and python scripts. Clean data reads were obtained from this step by removing reads with poly-N, 5’ adapter contaminants, ploy A/T/G/C, without 3’ adapter, the insert tag, and low quality.

Reads mapping to the reference sequence:
Using Bowtie, the small RNA tags were mapped to the reference sequence [1].

Known miRNA alignment:
miRBase20.0 was used as a reference to identify known miRNAs, by using the mapped small RNA tags to the reference. Then, modified mirdeep2 [2] and srna-tools-cli were used to obtain potential miRNA and draw secondary structures. Custom scripts were used to obtain the miR counts and base bias on the first position of identified miRNAs.

Novel miRNA prediction:
miREvo [3] and mirdeep2 [2] were used to predict novel miRNAs through exploration of the RNA secondary structures, the Dicer cleavage sites, and the minimum free energy of the previously unannotated small RNA tags. Simultaneously, custom scripts were used to obtain the miRNA counts and base bias on the first position of all identified miRNA.

Small RNA annotation summary:
To make every unique small RNA mapped to only one annotation, the following priority rule was followed: known miRNA > rRNA > tRNA > snRNA > snoRNA > repeat > gene > NAT-siRNA > gene > novel miRNA > ta-siRNA. The total rRNA proportion was used as a sample quality indicator.

miRNA editing analysis:
Positions 2~8 of mature miRNAs are called seed regions and are highly conserved. The miRNA targets may be different with potential changes in nucleotides in this region. In the analysis pipeline, miRNAs that may have base edits were detected by aligning all the sRNA tags to mature miRNA, allowing one mismatch.

Target gene prediction:
miRanda was used to predict the target genes of miRNAs.

Quantification of miRNA:
miRNA expression levels were estimated by TPM (transcript per million) using the following criteria [4]:
Normalization formula: Normalized expression = mapped read count/Total reads*1000000.

SUPPLEMENTAL RESULTS:

Supplementary Table S1. Quality Control (LncRNA and CircRNA):

	Control	HypoTH	HyperTH	HypoTH + T3
**Raw Reads**	40414882±2080208.475	41862489±306531.005	41492816.333±398483.857	54496711±12301952.995
**Clean Reads**	40052519.333±2050072.277	41114499±214828.350	40750017.333±399893.012	53719779±12072461.82
**Raw Data (G)**	12.133±0.636	12.566±0.088	12.433±0.12	16.366±3.667
**Clean Data (G)**	12±0.608	12.366±0.067	12.233±0.12	16.133±3.634
**Error (%)**	0.026±0.003	0.026±0.003	0.03±0	0.03±0
**Q20 (%)**	97.813±0.102	97.043±0.742	95.826±0.069	95.4±0.191
**Q30 (%)**	93.863±0.321	92.176±1.652	89.443±0.15	88.643±0.442
**GC (%)**	47.39±0.105	47.36±0.202	47.793±0.1	47.35±0.158

Values are mean ± SE. The details of the variables are described below:

- **Raw reads:** Reads count from the raw data, four rows as a unit, with statistics of reads count for every sequencing;
- **Clean reads:** Clean data are read count filtered from raw data. The statistical method is similar to raw reads. All the following analyses are based on clean data;
- **Raw data (Bases; Giga):** Base number of raw data. (Number of raw reads) * (sequence length), converting unit to G;
- **Clean data (Bases; Giga):** Base number of raw data after filtering. (Number of clean reads) * (sequence length), converting the unit to G;
- **Error rate (%):** Base error rate of whole sequencing;
- **Q20 (%):** Phred values greater than 20 base number contain the percentage of total bases. (Base number of Phred value > 20) / (Total base number)*100;
- **Q30 (%):** Phred values greater than 30 base number contain the percentage of total bases. (Base number of Phred value > 30) / (Total base number)*100;
- **GC content (%):** The percentage of G&C base numbers of total bases. (G&C base number) / (Total base number)*100.

Supplementary Table S2. Differentially expressed LncRNAs between HypoTH and Control groups.

Transcript	Gene Name	Gene ID	HypoTH Group Expression	Control Group Expression	Log2 (Fold Change)	p-value (Adjusted/ Corrected)
**NR_028422.1**	C130080G10Rik	100303644	0.891445	34.444394	-5.271979596	0.027635415
**XR_867569.1**	Gm40124	105244531	5.430553333	0	Inf	0.027635415
**TCONS_00004800**	**Synpo2**	**118449**	**1.056803667**	**0**	**Inf**	**0.027635415**
**TCONS_00009217**	1700030F04Rik	72263	1.307807667	0	Inf	0.027635415
**XR_380269.2**	Gm38485	102636048	1.170262333	0	Inf	0.027635415
**TCONS_00022369**	LOC118568312	118568312	375.3674317	0	Inf	0.027635415
**TCONS_00022949**	LOC118568312	118568312	27.22169733	0	Inf	0.027635415
**TCONS_00022951**	LOC118568312	118568312	20.39411167	0	Inf	0.027635415
**TCONS_00025736**	**mt-Rnr2**	**17725**	**16.98086167**	**0**	**Inf**	**0.027635415**
**TCONS_00025779**	-	XLOC_015265	12.45470933	0	Inf	0.027635415
**NR_045640.1**	Gm20199	100504377	1.138684333	0.103314	3.462260187	0.031451684
**NR_168272.1**	**Gm42067**	**105246846**	**1.114302333**	**4.448331667**	**-1.99712364**	**0.033344077**
**TCONS_00025777**	-	XLOC_015265	26.76265467	3.527620333	2.923454027	0.0355126
**XR_004934374.1**	Gm26944	102635071	0.987436667	0	Inf	0.048831628

The lncRNAs that are downregulated in HypoTH are given in blue and those that are upregulated are given in red. LncRNAs common within HypoTH vs. Control and HyperTH vs. HypoTH (below) comparisons are highlighted in bold.

Supplementary Table S3. Differentially expressed LncRNAs between HyperTH and HypoTH groups.

Transcript	Gene Name	Gene ID	HyperTH Group Expression	HypoTH Group Expression	Log2 (Fold Change)	p-Value (Adjusted/ Corrected)
NR_131913.1	Lincred1	100996933	0.373112333	2.218947333	-2.572193471	0.005399615
XR_380324.2	Gm36827	102640859	2.307737	0.259222667	3.154215038	0.007224164
TCONS_00011627	Chd9	109151	2.979505	0.92774	1.683280218	0.011849041
XR_871656.2	Gm36827	102640859	0	5.332116333	-Inf	0.011849041
NR_166471.1	Foxo6os	402730	7.148917	0.057763	6.95143522	0.011849041
NR_015601.1	Fam120aos	68128	0.710750333	4.032742667	-2.504346573	0.011849041
TCONS_00004800	Synpo2	118449	0	1.056803667	-Inf	0.011849041
TCONS_00025736	mt-Rnr2	17725	0	16.98086167	-Inf	0.011849041
XR_003950992.1	Gm52092	115488289	3.039186667	0.023409333	7.020457629	0.012025606
NR_045170.1	Gm10336	328186	53.12925967	12.389056	2.100440346	0.01313389
XR_880771.2	Gm42208	105247026	0.641111333	2.688013667	-2.067893656	0.01751405
NR_168057.1	D330025C20Rik	665054	0.547646333	2.623150667	-2.259984259	0.024594455
NR_040309.1	2310069G16Rik	69659	3.403704333	0.273044	3.639900361	0.027401014
TCONS_00013019	Gm28979	102633035	2.465509333	0.005822	8.726155159	0.029175858
XR_867957.2	Gm12701	105244634	1.073003	3.434677333	-1.678520463	0.031663793
NR_168272.1	Gm42067	105246846	4.790582333	1.114302333	2.104060319	0.032840751
NR_033503.1	E530011L22Rik	320301	0.085753667	1.51838	-4.146190725	0.033507249
XR_386629.3	Gm35268	102638787	4.213745667	0.927016	2.184437094	0.034294928
XR_001779774.2	Gm36827	102640859	3.463763	0.637568667	2.441687585	0.03699232

**NR_166847.1**	Gm11967	102639588	9.905372	0.943594	3.391973007	0.039509295
**NR_166742.1**	Gm41717	105246427	1.207936333	3.969141667	-1.716282639	0.042621026
**NR_045269.2**	Sp3os	100503849	0.949197667	3.116657667	-1.715219239	0.045071319
**XR_004941109.1**	LOC118568566	118568566	0.823173667	2.672833333	-1.699101144	0.047158879

LncRNAs common within HypoTH vs. Control and HyperTH vs. HypoTH comparisons are highlighted in bold. Those that are downregulated in HyperTH are given in blue and upregulated are given in red.

Supplementary Table S4. Differentially expressed LncRNAs between HypoTH+T3 and HypoTH groups.

Transcript	Gene Name	Gene ID	HypoTH+ T3 Group Expression	HypoTH Group Expression	Log2 (Fold Change)	p-Value (Adjusted/ Corrected)
**NR_015601.1**	**Fam120aos**	**68128**	**0.867212667**	**4.032742667**	**-2.217303615**	**0.01818002**
**XR_003950992.1**	**Gm52092**	**115488289**	**2.026253333**	**0.023409333**	**6.4355869**	**0.026987592**
**TCONS_00022791**	9630028I04Rik	319720	0.003526333	3.753323	-10.05578388	0.03616698
**XR_376121.3**	Gm31678	102633985	0.594435333	2.501163333	-2.073007495	0.048140492

LncRNAs common within HypoTH+T3 vs. HypoTH and HyperTH vs. HypoTH comparisons are highlighted in bold. Those that are downregulated in HypoTH+T3 are given in blue and those that are upregulated are given in red.

Supplementary Table S5. Differential expression of circRNAs between HyperTH and Control groups.

Gene	HyperTH group expression	Control Group Expression	log2(fold change)	p-Value (adjusted/ corrected)
**mmu_circ_ 4456**	60.14525446	1002.420908	- 4.058889532	0.0046353 62
**mmu_circ_ 5616**	80.19367261	1122.711417	- 3.807351926	0.0014045 21
**mmu_circ_ 2471**	60.14525446	741.7914716	- 3.624486861	0.0183179 77
**mmu_circ_ 3252**	60.14525446	621.5009627	- 3.369229916	0.0444205 73
**mmu_circ_ 1539**	140.3389271	982.3724895	- 2.807353026	0.0105407 53
**mmu_circ_ 799**	160.3873452	942.2756532	- 2.554587257	0.0159796 86
**mmu_circ_ 2045**	280.6778541	1323.195598	- 2.237038155	0.0063765 03
**mmu_circ_ 1548**	6876.607426	28208.12434	- 2.036341746	2.87E-47
**mmu_circ_ 2258**	320.7746904	1202.905089	- 1.906889743	0.0183179 77
**mmu_circ_ 960**	481.1620357	1423.437689	- 1.564783962	0.0401393 41
**mmu_circ_ 384**	521.258872	1503.631361	- 1.528378352	0.0401393 41

CircRNAs that are downregulated in HyperTH are given in blue.

Supplementary Table S6: sRNA statistics.

	Statistics of total reads mapped to ncRNA				Statistics of unique reads mapped to ncRNA			
**Types**	**Control**	**HypoTH**	**HyperTH**	**HypoTH+T3**	**Control**	**HypoTH**	**HyperTH**	**HypoTH+T3**
**rRNA**	5378602±793107.95	4294684±693529.87	1931337.66±208317.10	2569843.33±403666.28	54321.33±1454.24	44578±7973.23	28013.66±1076.90	28732.66±2516.73
**rRNA:+**	5378229±793135.09	4294575±693527.08	1931194±208323.52	2569727.66±403668.02	54217.33±1469.08	44527.33±7967.56	27954.66±1083.39	28683.33±2514.63
**rRNA:-**	373±31.39	109±8.33	143.66±6.77	115.66±21.40	104±15.04	50.66±6.94	59±8.19	49.33±10.33
**tRNA**	172074±16057.01	57565.33±25871.95	180905.66±61400.73	68582.33±17868.34	5362±173.30	3300±1219.04	2384±151.01	1632.33±214.79
**tRNA:+**	169937.33±16281.08	56488.33±25405.17	178670.66±61591.30	67864±17786.59	4912.66±179.14	3031.66±1100.99	2178.33±134.94	1521.66±198.04
**tRNA:-**	2136.66±262.50	1077±480.37	2235±209.69	718.33±102.23	449.33±7.67	268.33±118.51	205.66±27.85	110.66±17.29
**snRNA**	57910±8162.93	55011±17756.16	16260±586.06	13548.33±2093.37	6649.33±391.67	5823±1582.51	2107.66±45.84	2024±199.85
**snRNA:+**	57889.66±8161	54989±17750.46	16249.66±587.44	13538.66±2090.88	6634±391.33	5808.66±1577.68	2100±47.03	2018.33±198.33
**snRNA:-**	20.33±2.03	22±6.08	10.33±1.45	9.66±3.28	15.33±0.33	14.33±4.98	7.66±1.45	5.66±1.67
**snoRNA**	177770.33±6664.29	77646.66±23296.94	41398±739	41220±8455.35	18908.66±1117.95	14585.33±4259.05	5960.66±196.87	5453±611.58

**snoRNA :+**	177764±6665.53	77636.66±23293.46	41390±738.27	41216.33±8455.40	18903.66±1118.47	14576.66±4256.21	5956±197.05	5450±611.53
**snoRNA :-**	6.33±1.33	10±3.51	8±1.15	3.66±0.67	5±1.73	8.66±2.85	4.66±0.88	3±0.58

Statistics of total reads and unique reads mapped to ncRNA are presented. The small RNA reads were annotated with sequences from Rfam and matched reads were removed from the different small RNA types; rRNA: ribosomal RNA; tRNA: transfer RNA; snRNAs: Small nuclear RNAs; snoRNAs: Small nucleolar RNAs

Supplementary Table S7. Summary of the small RNA Quality Control.

Sample	Reads	Bases (G)	Error rate (%)	Q20 (%)	Q30 (%)	GC content (%)
**Control**	30587688±3245538.15	1.53±0.16	0±0	99±0	96±0	52±0
**HypoTH**	24307605±1473589.89	1.21±0.08	0±0	99±0	97±0	53±0
**HyperTH**	22084188.33±1100643.28	1.10±0.05	0±0	99±0	96±0	52±0
**HypoTH + T3**	23937723.33±2029995.81	1.19±0.10	0±0	99±0	97±0	52±0

- Reads: Statistics of the original sequence data;
- Bases: Sequence number multiplied by the length of the sequence, expressed in gigabase pairs (Gbp);
- Error rate: Sequencing error rate;
- Q20: Percentage of bases whose Phred values exceed 20;
- Q30: Percentage of bases whose Phred values exceed 30;
- GC content: G and C bases as a percentage of all bases.

Supplementary Figure S1. Morphometrics: Males.

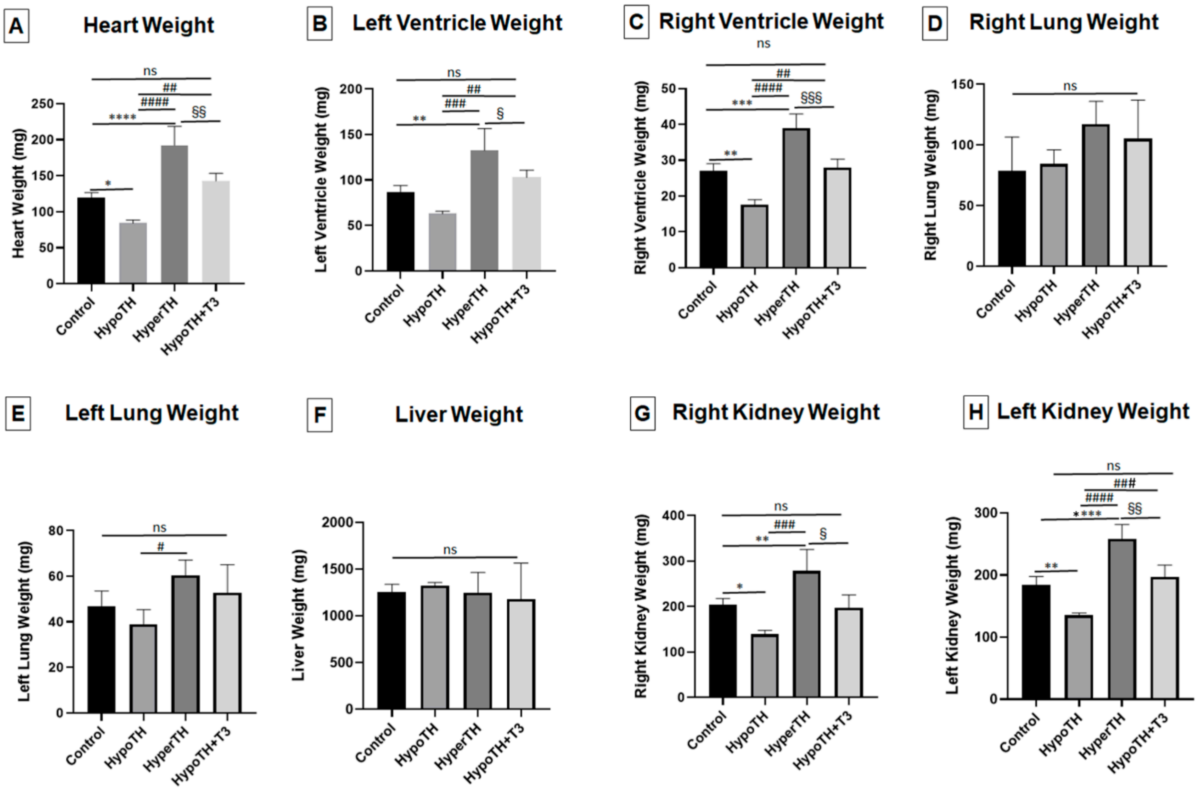

Supplementary Figure S1. Morphometrics (males): Alterations in (A) Heart, (B) Left Ventricle, (C) Right Ventricle, (D) Right Lung, (E) Left Lung, (F) Liver, (G) Right Kidney, and (H) Left Kidney weights when subjected to HypoTH, HyperTH and HypoTH + oral T3 in male mice. All values are mean ± standard deviation; n=3-5 per group. HyperTH: Hyperthyroidism; HypoTH: Hypothyroidism; T3: Triiodothyronine; *p<0.05, **p<0.01, ***p<0.001, ****p<0.0001 vs. controls; #p<0.05, ##p<0.01, ###p<0.001, ####p<0.0001 vs. HypoTH; §p< 0.05, §§p<0.01, §§§p<0.001 vs. HyperTH; ns: not significant.

Supplementary Figure S2. Morphometrics - Females:

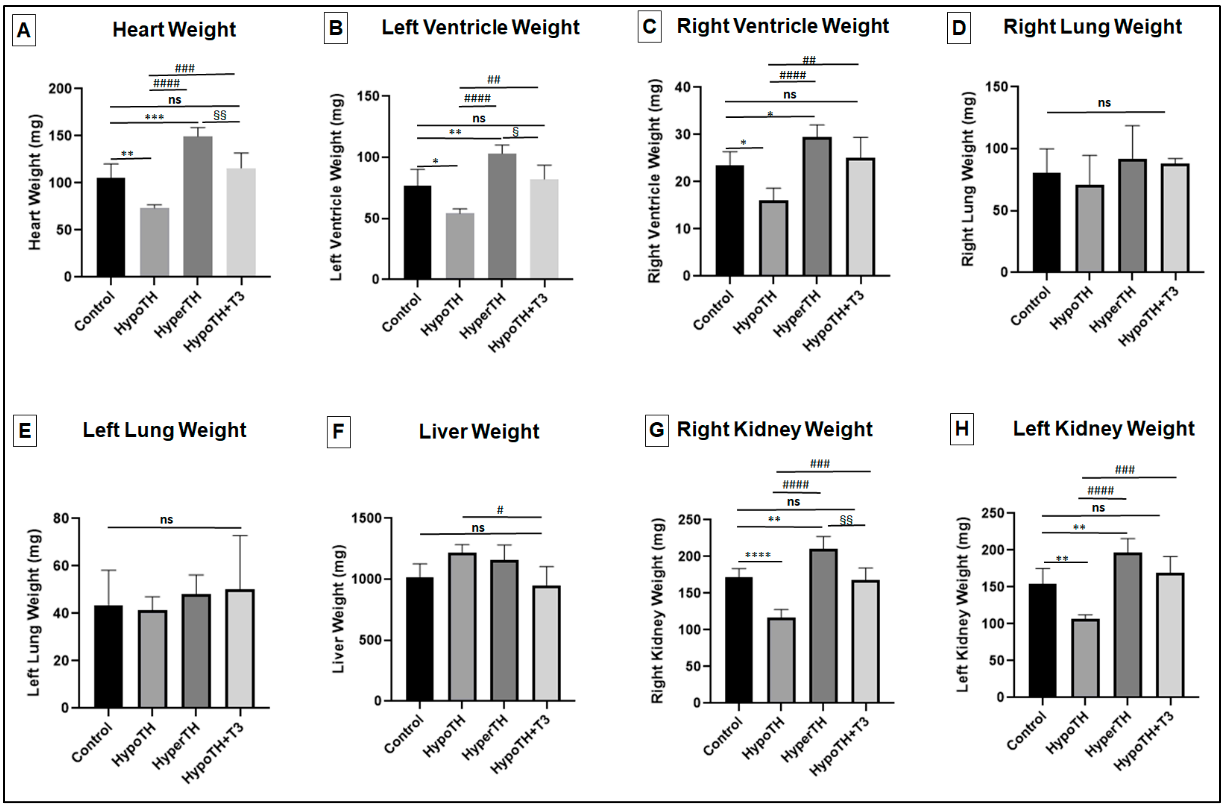

Supplementary Figure S2. Morphometrics (Females): Alterations in (A) Heart, (B) Left Ventricle, (C) Right Ventricle, (D) Right Lung, (E) Left Lung, (F) Liver, (G) Right Kidney, and (H) Left Kidney weights when subjected to HypoTH, HyperTH and HypoTH + oral T3 in female mice. All values are mean ± standard deviation; n=3-5 per group. HyperTH: Hyperthyroidism; HypoTH: Hypothyroidism; T3: Triiodothyronine; *p<0.05, **p<0.01, ***p<0.001, ****p<0.0001 vs. controls; #p<0.05, ##p<0.01, ###p<0.001, ####p<0.0001 vs. HypoTH; §p<0.05, §§p< 0.01 vs. HyperTH; ns: not significant.

Supplementary Figure S3. Significant impairment in cardiac inflammatory lncRNA expression under altered TH conditions.

`

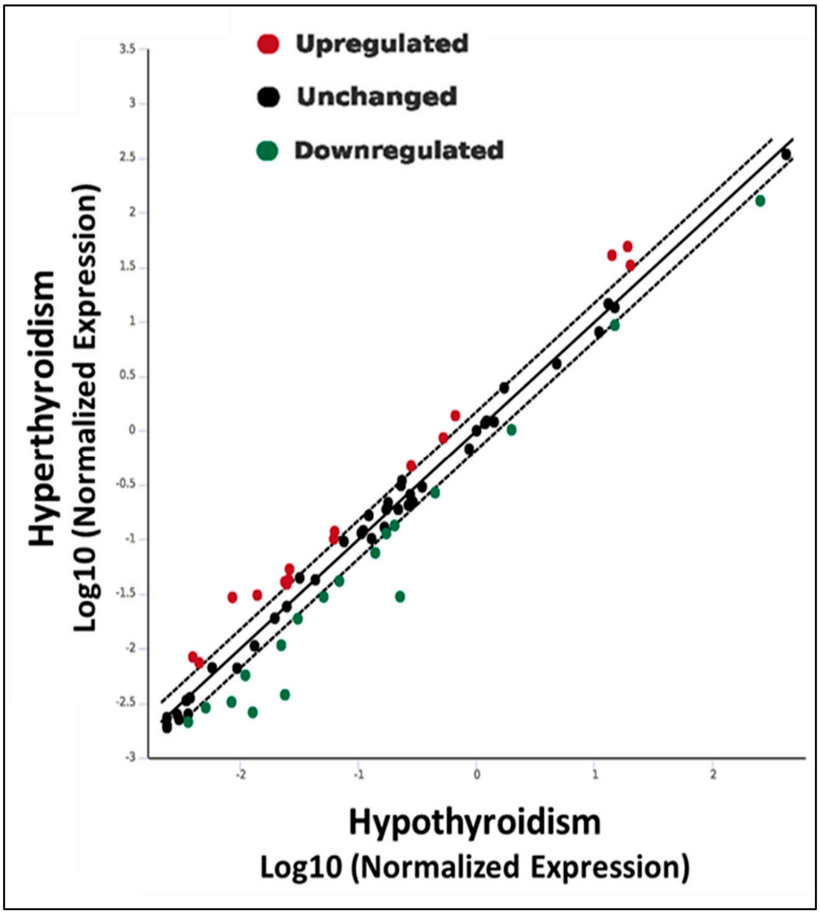

Supplementary Figure S3. Left ventricular lncRNA-specific qPCR arrays showed aberrant real-time expression with altered TH levels; n=4/group; >±1.5-fold change; p<0.05

Supplementary Figure S4. Differential expression heatmaps for noncoding and coding RNAs.

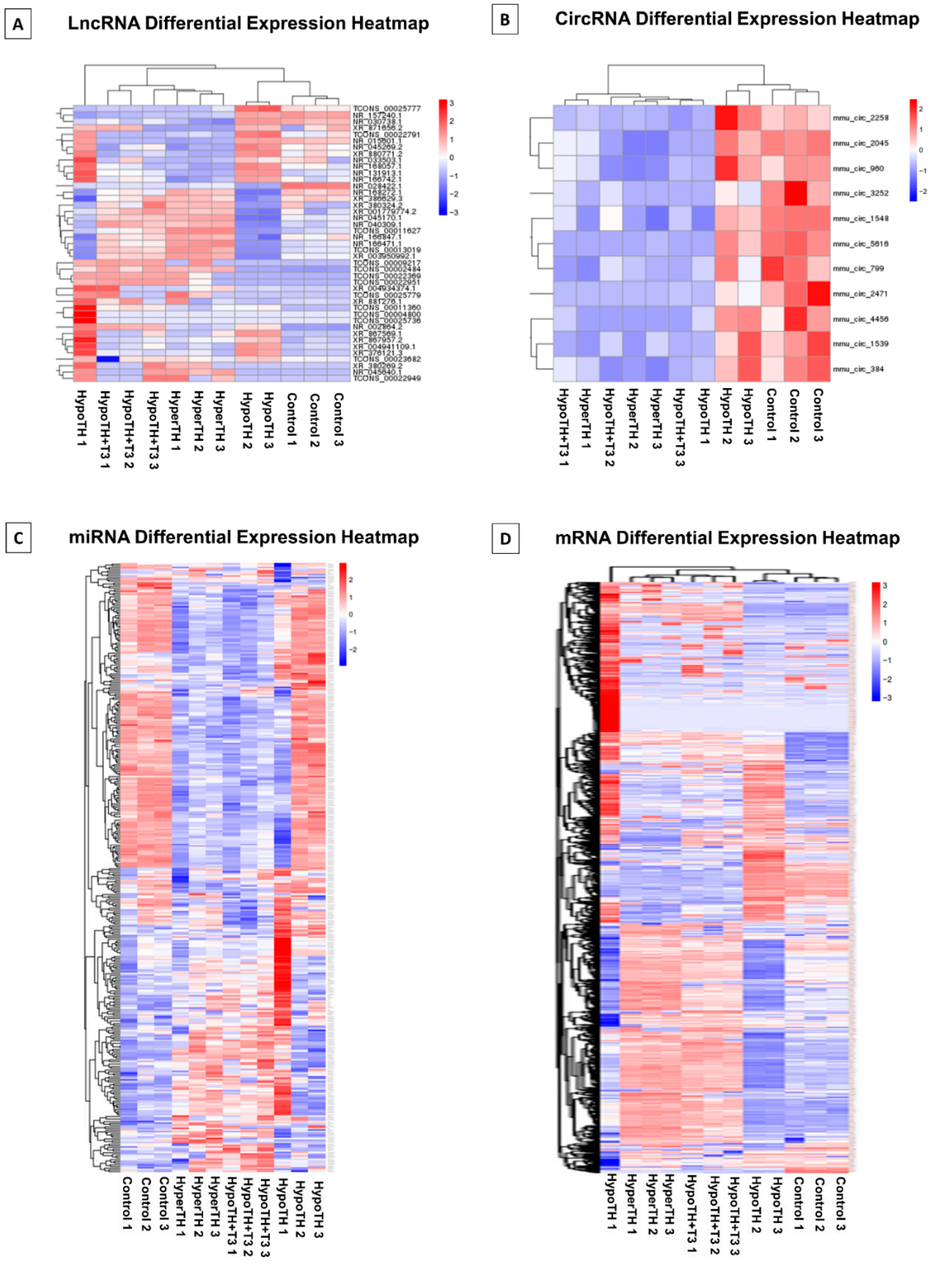

Supplementary Figure S4. Differential expression heatmaps (hierarchical clustering) of (A) lncRNAs, (B) circRNAs, (C) miRNAs, and (D) mRNAs. Rows indicate significantly differentially expressed RNAs and columns represent individual samples from all the four groups.

Supplementary Figure S5. GO Enrichment analysis of miRNA groupwise comparisons.

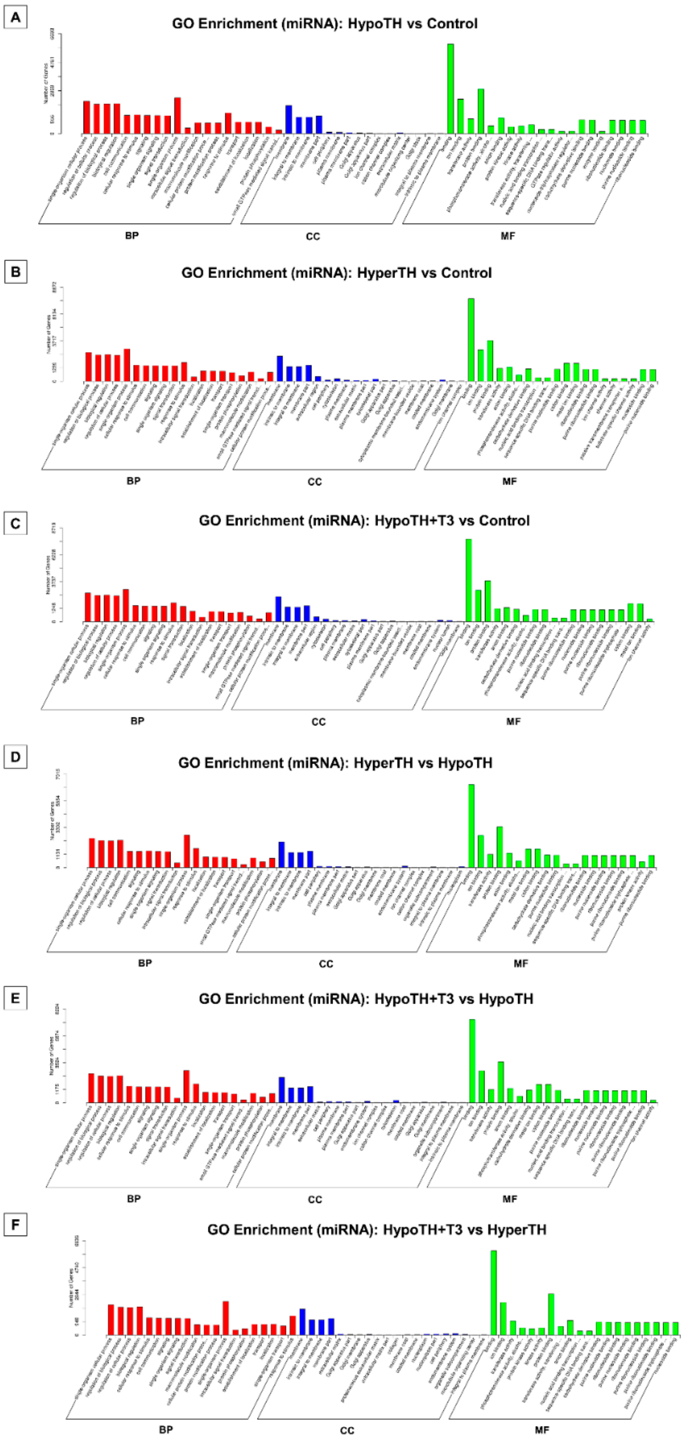

Supplementary Figure S5. GO Enrichment analysis of miRNA groupwise comparisons. Top 20 GO enrichment (Biological process, BP; Cellular compartment, CC; and Molecular function, MF) hits are plotted for **(A) HypoTH vs. Control; (B) HyperTH vs. Control; (C) HypoTH+T3 vs. Control; (D) HyperTH vs. HypoTH; (E) HypoTH+T3 vs. HypoTH; and (F) HypoTH+T3 vs. HyperTH group**. All the hits plotted here are statistically significant with p(adj/corr)<0.05**.**

REFERENCES:

1. Langmead B, Trapnell C, Pop M, Salzberg SL. Ultrafast and memory-efficient alignment of short DNA sequences to the human genome. Genome Biol. 2009;10(3):R25. Epub 2009/03/06. doi: 10.1186/gb-2009-10-3-r25. PubMed PMID: 19261174; PubMed Central PMCID: PMCPMC2690996.
2. Friedlander MR, Mackowiak SD, Li N, Chen W, Rajewsky N. miRDeep2 accurately identifies known and hundreds of novel microRNA genes in seven animal clades. Nucleic Acids Res. 2012;40(1):37-52. Epub 2011/09/14. doi: 10.1093/nar/gkr688. PubMed PMID: 21911355; PubMed Central PMCID: PMCPMC3245920.
3. Wen M, Shen Y, Shi S, Tang T. miREvo: an integrative microRNA evolutionary analysis platform for next-generation sequencing experiments. BMC Bioinformatics. 2012;13:140. Epub 2012/06/23. doi: 10.1186/1471-2105-13-140. PubMed PMID: 22720726; PubMed Central PMCID: PMCPMC3410788.
4. Zhou L, Chen J, Li Z, Li X, Hu X, Huang Y, et al. Integrated profiling of microRNAs and mRNAs: microRNAs located on Xq27.3 associate with clear cell renal cell carcinoma. PLoS One. 2010;5(12):e15224. Epub 2011/01/22. doi: 10.1371/journal.pone.0015224. PubMed PMID: 21253009; PubMed Central PMCID: PMCPMC3013074.
